# Single-Cell Transcriptomics Reveals Evolutionary Reconfiguration of Embryonic Cell Fate Specification in the Sea Urchin *Heliocidaris erythrogramma*

**DOI:** 10.1093/gbe/evae258

**Published:** 2024-11-26

**Authors:** Abdull J Massri, Alejandro Berrio, Anton Afanassiev, Laura Greenstreet, Krista Pipho, Maria Byrne, Geoffrey Schiebinger, David R McClay, Gregory A Wray

**Affiliations:** Department of Biology, Duke University, Durham, NC 27708, USA; Department of Biology, Duke University, Durham, NC 27708, USA; Department of Mathematics, University of British Colombia, Vancouver, BC, Canada V6T 1Z2; Department of Mathematics, University of British Colombia, Vancouver, BC, Canada V6T 1Z2; Department of Biology, Duke University, Durham, NC 27708, USA; School of Life and Environmental Sciences, Sydney University, Sydney, NSW, Australia; Department of Mathematics, University of British Colombia, Vancouver, BC, Canada V6T 1Z2; Department of Biology, Duke University, Durham, NC 27708, USA; Department of Biology, Duke University, Durham, NC 27708, USA

**Keywords:** evolution of development, life history evolution, scRNA-seq, transcriptional regulation, cell fate specification

## Abstract

Altered regulatory interactions during development likely underlie a large fraction of phenotypic diversity within and between species, yet identifying specific evolutionary changes remains challenging. Analysis of single-cell developmental transcriptomes from multiple species provides a powerful framework for unbiased identification of evolutionary changes in developmental mechanisms. Here, we leverage a “natural experiment” in developmental evolution in sea urchins, where a major life history switch recently evolved in the lineage leading to *Heliocidaris erythrogramma*, precipitating extensive changes in early development. Comparative analyses of single-cell transcriptome analysis (scRNA-seq) developmental time courses from *H. erythrogramma* and *Lytechinus variegatus* (representing the derived and ancestral states, respectively) reveal numerous evolutionary changes in embryonic patterning. The earliest cell fate specification events and the primary signaling center are co-localized in the ancestral developmental gene regulatory network; remarkably, in *H. erythrogramma*, they are spatially and temporally separate. Fate specification and differentiation are delayed in most embryonic cell lineages, although in some cases, these processes are conserved or even accelerated. Comparative analysis of regulator-target gene co-expression is consistent with many specific interactions being preserved but delayed in *H. erythrogramma*, while some otherwise widely conserved interactions have likely been lost. Finally, specific patterning events are directly correlated with evolutionary changes in larval morphology, suggesting that they are directly tied to the life history shift. Together, these findings demonstrate that comparative scRNA-seq developmental time courses can reveal a diverse set of evolutionary changes in embryonic patterning and provide an efficient way to identify likely candidate regulatory interactions for subsequent experimental validation.

**Significance**

Life histories of multicellular organisms are both astonishingly diverse and evolutionarily labile, yet the genomic and developmental bases for this component of biological diversity remain poorly understood. This study analyzes comparative developmental time courses of single-cell transcriptomes from two sea urchins with radically different life histories. The results reveal extensive and diverse evolutionary changes in development, including dissociations in the very earliest patterning events, temporal shifts in the timing of differentiation, altered proportions of larval cell types, and changes in interactions between specific transcription factors and target genes. Together, the results illustrate how single-cell transcriptomes can identify changes in development that are not evident from comparisons of morphology or bulk transcriptomic assays.

## Introduction

Most metazoan life cycles contain intermediate stages that are ecologically distinct from adults. In many clades, this has resulted in the evolution of contrasting anatomical, physiological, and behavioral traits between stages in the life cycle (Garstang 1928; Thorson 1950; Strathmann 1985; Nielsen 1998; Raff and Byrne 2006; Formery and Lowe 2023). Host-specific stages of parasites, insect larvae, amphibians, and diverse marine invertebrates are often so different from adults that they are unrecognizable from the earlier stages of the same life cycle. In some clades, the evolution of these intermediate stages is remarkably labile, such that closely related species with very similar adult morphology differ profoundly earlier in the life cycle. These cases likely reflect shifts in natural selection that operate on intermediate phases of the life cycle but not on adults. Numerous adaptations related to larval dispersal, feeding, predator avoidance, and abiotic factors have been documented. Yet, it remains largely unknown how developmental mechanisms known to pattern body organization at two distinct stages of the life cycle can become decoupled to allow effective responses to changing selective regimes.

The sea urchin genus *Heliocidaris* provides a valuable system for studying how developmental patterning becomes decoupled across life stages due to a combination of three salient features. First, the genus contains closely related species with highly divergent life histories and a known polarity of change. Second, the selective changes responsible for the life history shift are clear. And third, developmental mechanisms responsible for patterning the ancestral life history are well defined and organized into a developmental gene regulatory network (dGRN). Taken together, these features have made *Heliocidaris* a productive model for understanding genomic and developmental responses to large changes in stage-specific natural selection and their impact on life history evolution (Wang et al. 2020; Davidson et al. 2022a, 2022b; Devens et al. 2023).

**Fig. 1.**
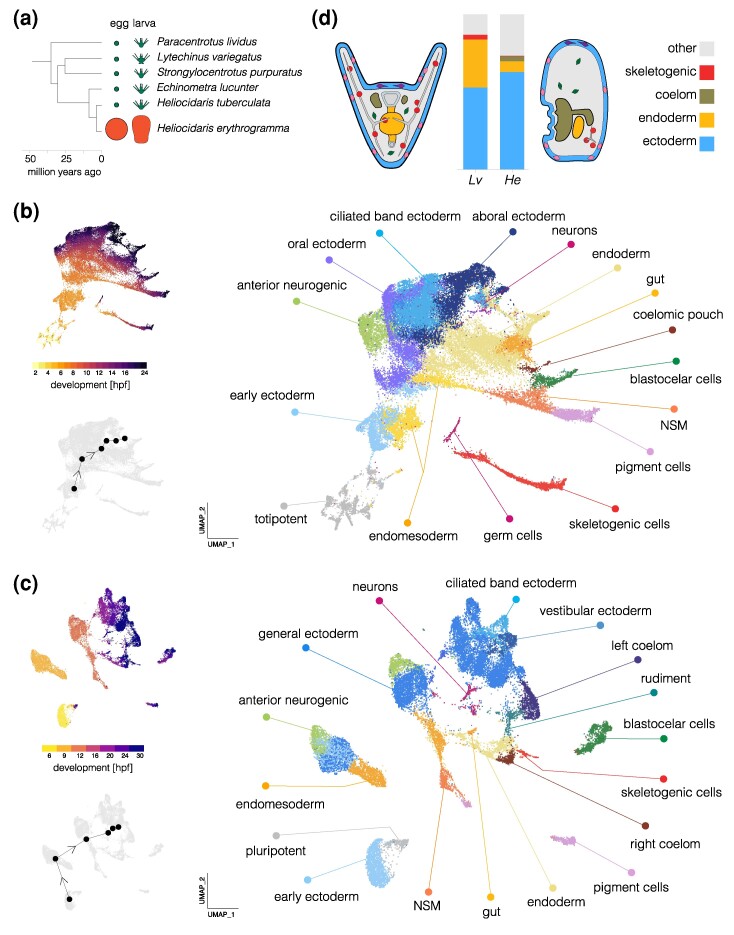
Comparison of single-cell developmental transcriptomes. a) Time tree of sea urchin species with high-quality reference genomes. Egg and larva sizes are approximately to scale; the ancestral life history is characterized by small eggs and feeding larvae (planktotrophy) and the derived life history by large eggs and nonfeeding larvae (lecithotrophy). Egg and larva sizes from Mortensen (1921), Emlet et al. (1987), Williams and Anderson (1975); topology from Láruson (2017); divergence times from Zigler et al. (2003) and Láruson (2017). b) UMAP plots of scRNA-seq developmental time course for *L. variegatus*. The large plot shows cells color coded by cluster and labeled according to inferred cell types; the two smaller plots show cells color coded by time point (upper) and the centroids of the six time points common to both species (lower plot). The earliest time point (2 hpf) cells are labeled “totipotent” based on blastomere separation experiments (Hörstadius 1973; Davidson et al. 1998). c) UMAPs of scRNA-seq developmental time course for *H. erythrogramma.* Organization parallels (b). For individual marker gene expression, see supplementary fig. S2, Supplementary Material online. Clusters in (b) and (c) are colored with the same encoding to facilitate comparison between species (some cell types are present only in one species or the other). Early blastomeres are not totipotent in this species (Henry and Raff 1990; Henry et al. 1990), but a distinct cluster of cells (labeled pluripotent) retains pluripotency into the larva (McDonald et al. 2024). d) Comparison of cell-type proportions and larval morphology. Proportions of four cell types in 24 hpf larvae (see supplementary tables S1 and S2, Supplementary Material online, for cell counts at all stages). Simplified diagrams of larvae are *not* to scale; colors match bar plot.

*Heliocidaris* illustrates how a shift in selective regimes can rapidly drive extensive changes in intermediate stages (Fig. 1a). Representing the ancestral state, *Heliocidaris tuberculata* produces small (∼100 µm diameter) eggs that develop into complex larvae that feed on phytoplankton for several weeks before achieving sufficient mass to complete metamorphosis. Representing the derived state, *H. erythrogramma* produces much larger eggs (∼430 µm diameter) in greatly reduced numbers, a classic life history trade-off (Stearns 1992). While this ∼100-fold increase in maternal provisioning might seem simple, its impact on other traits has been profound. The larva of *H. erythrogramma* is anatomically highly divergent from *H. tuberculata* (Williams and Anderson 1975; Fig. 1d). Unsurprisingly, it has lost the ability to feed: the gut and feeding structures are vestigial, presumably due to relaxed selection. In addition, metamorphosis occurs in just 5 d (Williams and Anderson 1975), a reduction of >75% in the duration of the premetamorphic phase of the life cycle. This enormous acceleration of early development seems unlikely to be the result of relaxed selection. Instead, the combination of high mortality in the plankton, coupled with greatly reduced fecundity due to the egg size-fecundity trade-off, likely imposes strong directional selection to reduce time to metamorphosis (Wray 2022). These striking differences in larval anatomy and life history evolved within the past ∼4 My, a short interval relative to their prior conservation of hundreds of millions of years (Fig. 1a).

In this study, we use single-cell transcriptome analysis (scRNA-seq) to investigate how extensive changes in larval anatomy and a >75% reduction in time to metamorphosis were achieved. We evaluated the presence and relative proportion of larval cell types, the timing of cellular differentiation, trajectories of transcriptional states as a proxy for cell lineages, and the co-expression of transcription factors and targets as indicators of specific regulatory interactions. Our results identify a broad delay in the divergence of transcriptional states during early development; changes in the timing, location, and order of cell fate specification and differentiation; and large shifts in the composition of cell types in the larva. In addition, some ancestral interactions within the dGRN are likely conserved in the derived life history, although most show changes in timing or location, and a few appear to have been lost entirely. Together, these analyses reveal evolutionary changes in embryonic patterning mechanisms and larval biology that were not apparent from morphological comparisons or from bulk RNA-seq analyses.

## Results

### Transcriptional States in Accurately Reflect the Evolution of Larval Morphology

We began by constructing an atlas of early development in *H. erythrogramma* for comparison with our previous analysis of *Lytechinus variegatus* that spanned early cleavage through early larva (Massri et al. 2021). To minimize confounds when comparing between species, our approach to generating data followed the earlier study as closely as possible, including rearing embryos at the same temperature, dissociating cells using only slightly species-optimized protocols, and employing the same generation of library construction and sequencing chemistry (see Materials and Methods). We collected seven time points of *H. erythrogramma* development from a single cross of outbred adults, from late cleavage (6 h postfertilization; hpf) through early larva (30 hpf; see Materials and Methods). We mapped reads to reference genomes for the two species that were generated in parallel from gDNA extraction through library preparation and sequencing to assembly and annotation (Davidson et al. 2020, 2022b), thus minimizing confounds that can arise from mapping reads to reference genomes with different quality and completeness. We recovered sequences from a total of 23,169 cells after filtering (average ∼3,310 cells/time point). Across samples, we obtained reads from ∼1,000 genes/cell and ∼2,000 to 3,000 Unique Molecular Indexes (UMIs) per cell (supplementary fig. S1, Supplementary Material online). The number of genes detected per cell drops across the stages sampled, likely reflecting differentiation.

We carried out clustering and dimensional reduction on the scRNA-seq time courses from both species in parallel using the same approach. Briefly, we used Seurat v4 to filter single cells, then normalized using SCTransformv2 and applied principal component analysis, followed by finding nearest neighbors and clusters, and finally Uniform Manifold Approximation and Projection (UMAP) dimensional reduction. We carried out analyses with resolutions 0.5, 1.0, 2.0, and 3.0 and found 18, 23, 36, and 48 clusters, respectively, for *H. erythrogramma*; for purposes of visualization, we collapsed these to 17 cell types based on marker genes following Massri et al. (2021). The resulting UMAPs (Fig. 1b and c) are colored by cell cluster (larger plot) and developmental time (upper inset). In both cases, early stages are in the lower left (hot colors) and development proceeds up and right to later stages (dark colors); small gray UMAPs show centroids of stages common to both species. As expected, the spread of points increases during development as cells take on distinct transcriptional states. The distribution of cells is nearly continuous for *L. variegatus*, while that of *H. erythrogramma* is more fragmented, likely due to less dense sampling (hourly in *L. variegatus* and every 3 h in *H. erythrogramma*). To identify cell clusters in *H. erythrogramma*, we drew on published in situ hybridization studies and dGRN genes with conserved expression in specific cell types to annotate clusters with provisional identities (supplementary fig. S2, Supplementary Material online; see Massri et al. 2021, for marker genes and supporting literature).

Several cell clusters in *H. erythrogramma* larvae (24 and 30 hpf) could be confidently assigned to a corresponding cluster in *L. variegatus* (24 hpf): pigment cells, blastocoelar (immune) cells, skeletogenic cells, endoderm, coelomic pouch, ciliated band ectoderm, generalized ectoderm, anterior neurogenic ectoderm, and neurons (supplementary fig. S2, Supplementary Material online). Each was previously shown to be present in early larvae of *H. erythrogramma* based on morphology and marker genes (Mortensen 1921; Williams and Anderson 1975; Parks et al. 1988; Bisgrove and Raff 1989; Wilson et al. 2005; Love et al. 2008; Koop et al. 2017).

Several differences in the UMAPs reflect the highly derived morphology of the nonfeeding *H. erythrogramma* larva relative to the ancestral feeding larvae of most sea urchins, including *L. variegatus* (Mortensen 1921; Williams and Anderson 1975; Wray and Raff 1989). Two clusters present in the 24 h larva of *L. variegatus* appear to be absent from *H. erythrogramma*: primary germ cells and stomodeum (mouth). The absence of germ cells is consistent with the evolutionary loss in *H. erythrogramma* of unequal cleavage divisions that found the primary germ cells lineage in the ancestral state (Pehrson and Cohen 1986; Oulhen et al. 2019). The lack of stomodeal cells corresponds to the absence of a larval mouth (Mortensen 1921; Williams and Anderson 1975).

Conversely, some cell clusters in *H. erythrogramma* are not present *L. variegatus* up to 24 hpf. These clusters are more challenging to identify: their apparent absence in well-studied species with the ancestral life history means that there are no described marker genes that can be used to identify cell types in *H. erythrogramma*. One of these clusters remains in close proximity in UMAP space to the single cluster of 6 hpf cells even at 30 hpf (labeled “pluripotent” in Fig. 1c). In a separate study, we show that these cells give rise to several different cell types in at least two germ layers in the late larva and juvenile rudiment of *H. erythrogramma* (McDonald et al. 2024). No corresponding cluster is evident in *L. variegatus* (Fig. 1b). These cells thus likely represent population pluripotent cells that are evolutionarily novel in the *H. erythrogramma* embryo. Other clusters uniquely present in *H. erythrogramma* likely consist of cells that contribute to the adult body (vestibular ectoderm and rudiment, Fig. 1c), which develops much earlier in *H. erythrogramma* (Williams and Anderson 1975; Wray and Raff 1989; Koop et al. 2017). We also found that ectoderm in *H. erythrogramma* expresses markers for the oral and aboral territories present ancestrally; consistent with prior studies based on in situ hybridization (Haag and Raff 1998; Love and Raff 2006; Koop et al. 2017); however, ectodermal gene expression is organized into a somewhat different set of clusters (Figs. 1c;  supplementary fig. S3, Supplementary Material online) that are not obvious 1:1 homologues of ectodermal territories in *L. variegatus*.

Substantial differences in the proportions of some cell types are also apparent. Because dissociation protocols can result in biased representation of cell types in scRNA-seq libraries, such findings need to be interpreted with caution. We therefore examined these results in light of prominent morphological differences between the larvae of two species, and highlight three differences that likely reflect true evolutionary changes in cell-type proportions in early larvae (Fig. 1d; supplementary tables S1 and S2, Supplementary Material online). First, endoderm makes up a much smaller fraction of cells in *H. erythrogramma* than *L. variegatus* (6.8% vs. 31.2%), consistent with its reduced and undifferentiated endoderm (Williams and Anderson 1975; Love et al. 2008). Second, the coelomic pouches contain many more cells in *H. erythrogramma* than *L. variegatus* (3.4% vs. 0.01%). This likely reflects the greatly accelerated development of the imaginal adult rudiment, a large fraction of which is composed of the left coelom (Williams and Anderson 1975; Wray and Raff 1989). Third, a much smaller proportion of skeletogenic cells are present in *H. erythrogramma* than *L. variegatus* (0.8% vs. 2.9%). This is consistent with its greatly reduced larval skeleton (Emlet 1995) and antibody localization of the marker protein Msp130 (Parks et al. 1988). The differences in proportions of the last two cell types are so extreme that they are barely visible in one or the other species in Fig. 1d.

### Cell Fate Specification is Broadly Delayed in *H. erythrogramma*

Examination of the UMAPs at earlier stages of development reveals additional differences (Fig. 1b and c). We first identified cell clusters corresponding to two functionally significant territories: the anterior neurogenic domain and the primary signaling center. In the ancestral state, the anterior neurogenic domain is located at the animal pole and develops into the primary sensory organ of the larva (Angerer et al. 2011). The anterior neurogenic domain is clear in *H. erythrogramma*, with overlapping expression of dGRN regulators *six3*, *foxQ2*, *nkx3.2*, *zic1*, and *acsc* (supplementary fig. S3, Supplementary Material online). The primary signaling center is located at the vegetal pole and produces ligands that initiate a cascade of signaling events that pattern the animal-vegetal axis (Davidson et al. 1998; McClay 2011). In the ancestral state, the primary signaling center is established ∼3 hpf in the precursors of the skeletogenic cells; they express genes encoding ligands, including *wnt8*, *wnt1*, and *delta* (Sherwood and McClay 1999; Sweet et al. 2002; Wikramanayake et al. 2004; Wei et al. 2012). In *H. erythrogramma*, these genes are expressed together within a single cluster (Fig. 1c; supplementary fig. S2, Supplementary Material online), but beginning much later (6 to 9 hpf). These cells also express *foxA* and other markers of endoderm (supplementary fig. S2, Supplementary Material online). These results suggest that the primary signaling center has become physically separated from the specification of skeletogenic cells, a surprising reorganization of pivotal early patterning events in the embryo.

Some other clusters in the *H. erythrogramma* embryo could not be confidently assigned to corresponding clusters in *L. variegatus*. The earliest time point sampled (6 hpf) consists of a single cluster lacking any distinctive transcriptional signature, provisionally labeled “early ectoderm” in Fig. 1c because it expresses ectodermal markers but not markers of any specific ectodermal territory. (The adjacent cluster, labeled pluripotent, consists of cells from later stages.) Of note, there is no indication of an early population of either skeletogenic mesenchyme or germ cells in *H. erythrogramma*. This represents a striking difference, as these are the first two cell types specified in the ancestral state, and each shows a distinct transcriptional state by 4 hpf in *L. variegatus*. Not until 16 hpf in *H. erythrogramma* is a population of skeletogenic cells evident, a remarkable delay relative to the ancestral state. While a distinct germ cell cluster is clear in *L. variegatus* (Fig. 1b), at no time is a distinct group of cells expressing germ cell markers evident in *H. erythrogramma*. *nanos2* and *vasa*, two early regulators of germ cell species with feeding larvae (Juliano et al. 2010; Oulhen et al. 2019), are co-expressed at 9 and 12 hpf in *H. erythrogramma* in the presumptive endoderm (supplementary fig. S2, Supplementary Material online), but expression disappears at later stages. These observations suggest that some early fate specification events are delayed in *H. erythrogramma* relative to *L. variegatus*.

**Fig. 2.**
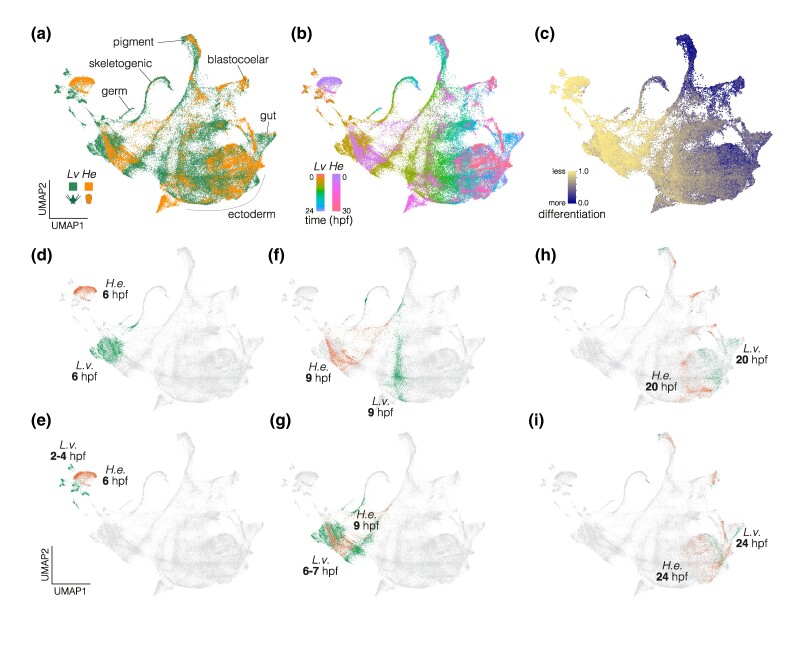
Integrated single-cell transcriptomes. Developmental transcriptomes of 1:1 orthologs in the two species were integrated using CCA (Butler et al. 2018), followed by dimensionality reduction. a) UMAP color coded by species. Relevant cell clusters are indicated. b) UMAP color coded by developmental time point. The earliest stages sampled are at the upper left, and development generally progresses down and to the right. c) UMAP color coded by degree of differentiation based on CytoTRACE (Gulati et al. 2020). Differentiation tracks developmental time points, with the lowest scores corresponding to the earliest time points and the highest scores corresponding to clusters of differentiated cells. d–g) Cells from 6 and 9 hpf *H. erythrogramma* do not overlap *L. variegatus* cells from the same time points (top UMAPs), and instead overlap earlier time points (bottom UMAPs). h, i). Cells from 20 and 24 hpf *H. erythrogramma* converge on *L. variegatus* cells from the same time points.

To better understand these differences quantitatively, we systematically analyzed the timing of transcriptional states in the two species. We integrated reads from 1:1 orthologs of *L. variegatus* and *H. erythrogramma* using canonical correlation analysis (CCA; Butler et al. 2018) prior to dimensional reduction. The resulting UMAP is presented colored by species (Fig. 2a) and by time (Fig. 2b). In these plots, cells from the two species broadly overlap in dimensions 1 and 2. Developmental time in both flows in parallel, and differentiated cells are closely juxtaposed; examples include pigment and blastocoelar cells (Fig. 2a). Differences in cell proportions are also evident; for instance, fewer gut and skeletogenic cells from *H. erythrogramma* are present. We also examined the integrated object using CytoTRACE (Gulati et al. 2020), which estimates the overall degree of differentiation in each cell without reference to information about developmental time (Fig. 2c). Transcripts representing undifferentiated cells are enriched in the upper left; a progressive increase in differentiation tracks developmental stages down and to the right. The most differentiated cells are located at the top and right periphery of the UMAP, corresponding to the latest stages sampled.

Despite broad overlap in cells between the two species, prominent temporal shifts are apparent. For example, cells from 6 hpf, the earliest time point sampled in *H. erythrogramma*, are not located near 6 hpf cells from *L. variegatus* in UMAP space (Fig. 2d), and instead overlap 2 to 4 hpf cells from *L. variegatus* (Fig. 2e). Similarly, 9 hpf cells from the two species do not overlap (Fig. 2f), and those from *H. erythrogramma* are located between 6 and 7 hpf cells from *L. variegatus* (Fig. 2g). At 20 hpf, cells in the two species are closer, but still largely distinct in their distributions (Fig. 2h) and not until 24 hpf is overlap extensive (Fig. 2i). Together, these observations suggest that developmental transcriptomes follow similar trajectories in the two species, but on rather different schedules: *H. erythrogramma* transcriptomes are initially delayed by 2 to 3 h relative to *L. variegatus*, and only come into alignment at ∼24 hpf.

**Fig. 3.**
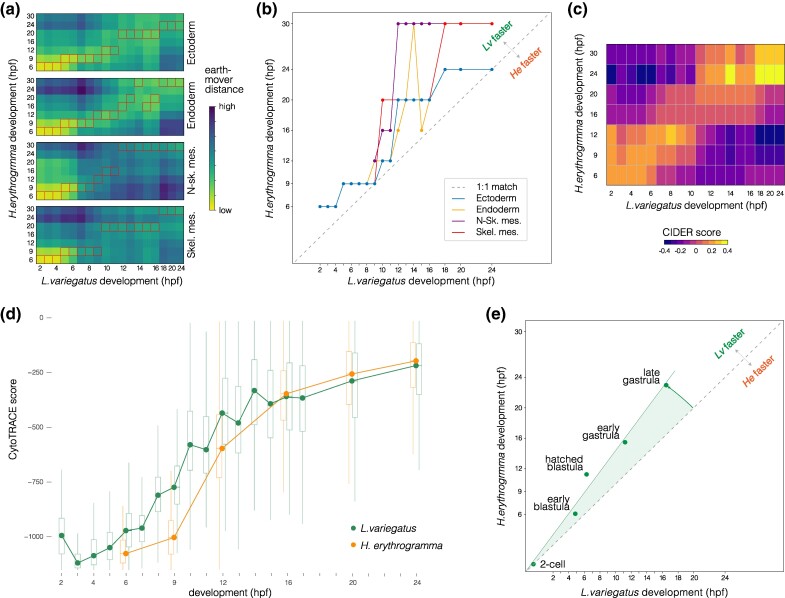
Temporal shifts in transcriptomes. a) Heat maps showing degree of similarity among scRNA-seq transcriptomes for four embryonic cell lineages in the two species. Assignment of cells to lineages is based on optimal transport (see Materials and Methods). Boxes highlight the most similar time points. N-sk. Mes., nonskeletogenic mesenchyme; Skel. Mes., skeletogenic mesenchyme. b) Line plots of the most similar time points in (a) reveal an overall delay in *H. erythrogramma*, with most points above the line defined by a slope of 1. c) Heat map showing degree of similarity for the entire scRNA-seq transcriptome at each stage based on CIDER (Hu et al. 2021). The first few time points in *H. erythrogramma* are most similar to earlier time points in *L. variegatus*. d) Plot of CytoTRACE scores (Gulati et al. 2020) during development show an initial delay in differentiation in *H. erythrogramma*, followed by convergence after ∼16 hpf. e) Line plot showing developmental time of morphogenetic events for comparison. Again, there is an overall delay in *H. erythrogramma*.

To investigate these evolutionary shifts in timing quantitatively, we first used Waddington OT, an approach that implements an optimal transport algorithm to generate a model of transitions between distinct transcriptional states during development (Schiebinger et al. 2019). Based on this model, we computed separate transcriptional trajectories for four cell types: endoderm, ectoderm, nonskeletogenic mesenchyme (lineage leading to blastocoelar and pigment cells), and skeletogenic mesenchyme (lineage leading to skeletogenic cells). We then measured the overall distance between transcriptomes in the two species within each cell lineage (Fig. 3a). The most similar time points are indicated by boxes and plotted in 1:1 aspect ratio in Fig. 3b. Points lie predominantly above a line of slope = 1 in Fig. 3b in all four cell lineages, indicating that progression through transcriptional states is broadly delayed throughout embryonic development in *H. erythrogramma*. We also used a meta-clustering method implemented in CIDER (Hu et al. 2021) to measure overall, rather than lineage-specific, similarity of transcriptomes (Fig. 3c) and plotted mean CytoTRACE scores at each time point (Fig. 3d). Both approaches indicate that transcriptional states in *H. erythrogramma* lag behind those in *L. variegatus*. This broad transcriptional delay is consistent with a comparison of developmental stages based on morphology, which also shows a delay in *H. erythrogramma* (Fig. 3e). Because the two species were reared at the same temperature, these rate differences are likely genetically based.

### Differentiation is Broadly Delayed in *H. erythrogramma*

**Fig. 4.**
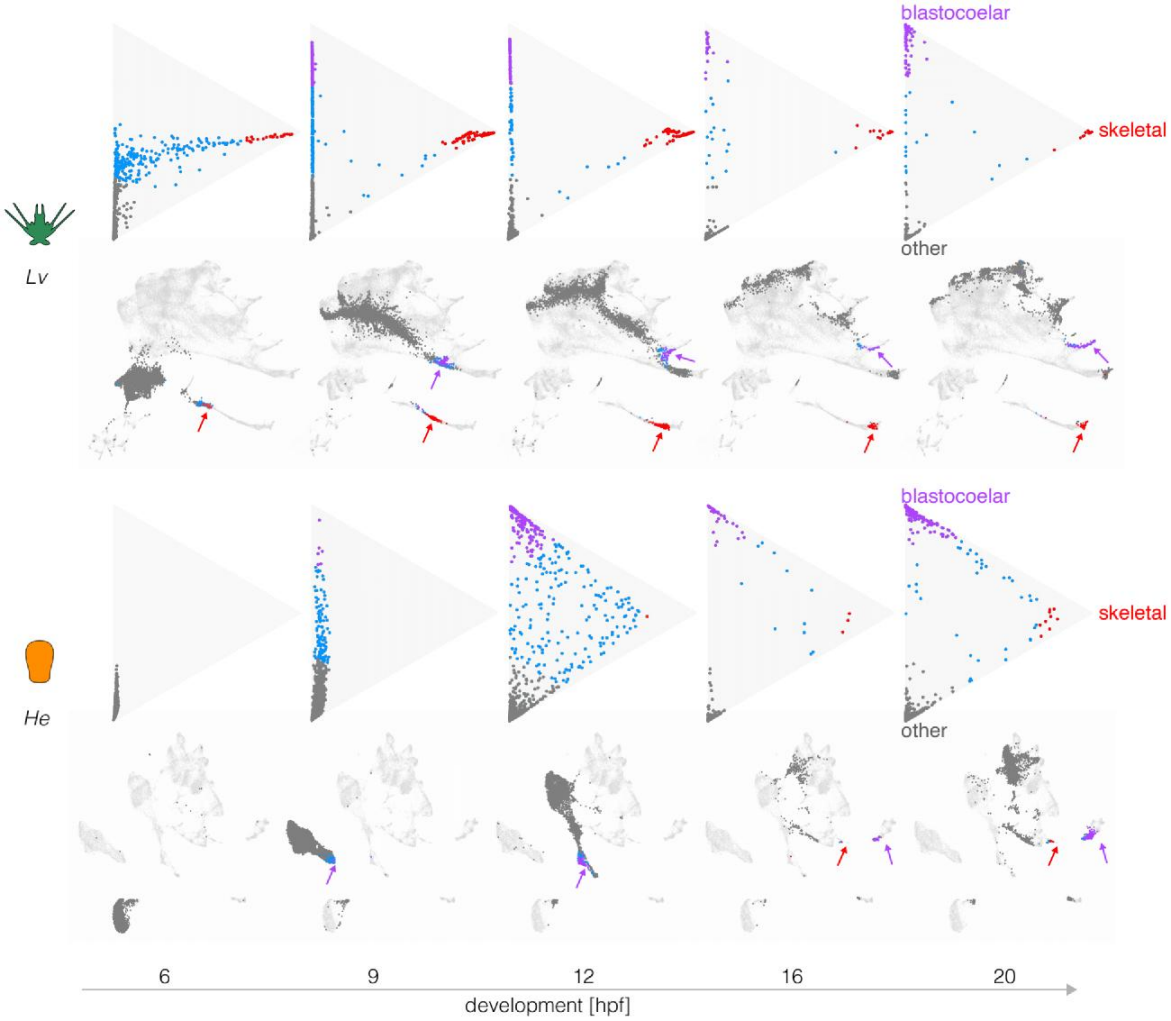
Evolutionary changes in timing of differentiation. Optimal transport was used to predict the likely fate for each cell at five stages, based on transcriptomes at 24 hpf (see Materials and Methods). Triangle plots show transcriptomes predictive of blastocoelar cell, skeletogetogenic cell, or any other cell fate near the top, right, and bottom vertices, respectively; cells with undifferentiated transcriptomes occupy the center. Corresponding UMAPs are shown below. Note the much earlier differentiation of skeletogenic cells in *L. variegatus* and the slightly earlier differentiation of blastocoelar cells in *H. erythrogramma*. See text for additional interpretation.

To better understand the timing of expression changes during differentiation, we plotted transcriptional trajectories toward defined differentiated cell states (Fig. 4;  supplementary fig. S4, Supplementary Material online) following Schiebinger et al. (2019). In these plots, each dot corresponds to a cell, with those nearest the upper and right vertices representing 70% probability of differentiating into a blastocoelar or skeletogenic cell fate, respectively; those near the lower vertex represent transcriptomes predictive of other cell fates and central cells are uncommitted. The triangle is a flattened projection of a high-dimensional space, with the location of each cell indicating the degree of similarity between its transcriptome and that of two specific differentiated states (top and right apexes) and all other differentiated states (bottom apex). The transcription profile that defines each apex is based on gene expression at 24 h, when many cell types in *L. variegatus* are approaching a fully differentiated state (Massri et al. 2021). Immediately below each triangle plot is a UMAP showing the location of the same cells.

Figure 4 shows that in *L. variegatus*, many cells take on a transcriptional state predictive of differentiating into a skeletogenic cell as early as 6 hpf (top left triangle plot, red dots). It is not until 9 hpf that a subset of cells are predicted to differentiate into blastocoelar cells (purple dots), consistent with the order of differentiation of these cells in *L. variegatus* (McClay 2011).

Several informative differences are evident in *H. erythrogramma*. First, the order of differentiation is reversed. Blastocoelar cell transcriptomes appear well before those of skeletogenic cells in *H. erythrogramma*, whereas skeletogenic cells begin differentiating long before blastoceolar cells in *L. variegatus*. This appears to be due primarily to a shift in skeletogenic cell differentiation, since blastocoelar cells are evident at 9 hpf in both species, while skeletogenic cells appear ∼6 h later in *H. erythrogramma* than *L. variegatus*. Second, at 20 hpf many dots remain far from any apex in *H. erythrogramma*, but most dots are at or near an apex in *L. variegatus*. This indicates that more cells remain uncommitted to any specific cell fate in the early larva of *H. erythrogramma* than that of *L. variegatus*. Finally, the degree of skeletogenic cell differentiation differs between species. None of the skeletogenic cell transcriptomes reaches the apex in *H. erythrogramma*, while many do so in *L. variegatus*, and they begin to arrive much earlier in development (9 hpf). A rather different pattern is seen with blastocoelar cells, where many reach the apex in both species, and this begins earlier in *H. erythrogramma* (12 hpf) than *L. variegatus* (20 hpf).

Analysis of other cell types reveals additional evolutionary changes in the timing and degree of differentiation, as well as an example of conservation in timing (supplementary fig. S4, Supplementary Material online). Two cell types are noteworthy because changes in their rate of differentiation may be related to the life history shift. Endoderm shows a particularly large delay in the onset of differentiation in *H. erythrogramma*: endodermal cells are evident at 9 hpf in *L. variegatus*, but even at 20 hpf, none are present in *H. erythrogramma* (supplementary fig. S4a and b, Supplementary Material online). Coelom shows a less dramatic delay in initial differentiation in *H. erythrogramma*, but the number of coelomic cells in *H. erythrogramma* overtakes those in *L. variegatus* (supplementary fig. S4a, Supplementary Material online). In contrast, blastocoelar and pigment cells show similar overall trajectories in the two species (supplementary fig. S4c, Supplementary Material online), indicating that the pace of differentiation within some cell lineages remains relatively unchanged.

Together, these analyses reveal a complex mosaic of evolutionary changes in the timing, order, and degree of differentiation among the two species. While the onset of differentiation is generally delayed in *H. erythrogramma*, individual cell types have evolved in distinct ways: blastocoelar cells differentiate earlier in *H. erythrogramma* relative to *L. variegatus*, some other cell types are delayed to different degrees (coelom less so than skeletogenic cells and gut), and some differentiate at about the same time (pigment cells).

### The Order of Cell Fate Specification is Altered in *H. erythrogramma*

The evolutionary differences in the timing of differentiation noted above are consistent with cell lineage tracing studies in *H. erythrogramma* (Wray and Raff 1989, 1990). However, those studies also suggested that the order of cell fate specification decisions might differ. We therefore reconstructed transcriptional trajectories during development (Chen et al. 2018; Kester and van Oudenaarden 2018; Forrow and Schiebinger 2021) based on the optimal transport model (Schiebinger et al. 2019; Forrow and Schiebinger 2021). The results are shown in supplementary fig. S5, Supplementary Material online. While previous analyses focused on evolutionary differences in timing (Figs. 3 and 4), the goal of this analysis was to identify possible evolutionary differences in the topology of the early cell lineage in *H. erythrogramma*.

As a positive control, we first evaluated how well transcriptional trajectories based on scRNA-seq data recapitulate actual cell lineages using the published *L. variegatus* time course (Massri et al. 2021), where the cell lineage is well defined by independent methods (McClay 2011). In the resulting directed graph (supplementary fig. S5a, Supplementary Material online), nodes correspond to cell clusters and edges connect nodes to their inferred “ancestor” (darker edges indicate higher confidence). This graph contains several features consistent with published analyses of embryonic cell lineages in *L. variegatus* and other sea urchins with feeding larvae (Hörstadius 1973; Pehrson and Cohen 1986; Cameron et al. 1987, 1990; Ruffins and Ettensohn 1996; Martik and McClay 2017). In particular, skeletogenic and primary germ cells diverge very early; pigment and coelomic cells share a common source population that is distinct from other endomesodermal cells; coeloms, stomodaeum, and gut share a common origin; and neurons derive from both gut and from the anterior neurogenic domain.

However, some inconsistencies are present. Most notably, there is no cluster that corresponds to the four micromeres, the direct ancestors of the skeletogenic and primary germ cell clonal founders. This is likely because cell lineage-specific zygotic transcription is extremely limited at the time the micromeres are present (Ernst et al. 1980) and thus overwhelmed by uniformly distributed maternal transcripts. In addition, the germ cell lineage is discontinuous and shows a late contribution from ectoderm; these are artifacts that are likely due to their tiny number (8 cells) in proportion to the rest of the embryo at later stages (>1,000 cells at 24 hpf; Pehrson and Cohen 1986). Finally, it should be noted that the ancestor-descendant linkages are in general rather noisy, with several spurious connections. Despite these inconsistencies, the overall topology of the graph resembles the cell lineage as defined by more direct forms of evidence (see citations above).

We then applied the same approach to the *H. erythrogramma* scRNA-seq time course (supplementary fig. S5b, Supplementary Material online). This graph shares some similarities with that of *L. variegatus*: gut and coeloms are closely related, as are ectodermal territories, including the anterior neurogenic domain. However, several other features are notably different. In *H. erythrogramma*, skeletogenic cells are among the last cell clusters to become transcriptionally distinct and are most closely related to blastocoelar cells, while in *L. variegatus*, they are one of the first to become transcriptionally distinct and are not related to blastocoelar cells. In addition, pigment cells in *H. erythrogramma* are not closely related to blastocoelar cells and become transcriptionally distinct well before they do, while in *L. variegatus*, pigment cells and blastocoelar cells derive from a unique common precursor population and simultaneously diverge transcriptionally (Massri et al. 2021). These differences are consistent with the triangle plots (Fig. 4 and supplementary fig. S4, Supplementary Material online). They also imply evolutionary changes in the temporal order and spatial location of fate specification among mesodermal cell lineages (see Discussion).

Other differences in the two graphs are associated with structures or cell types that are present in the larva of one species but not the other. This is apparent in the ectoderm, which is organized anatomically and transcriptionally into somewhat different territories in *H. erythrogramma* relative to the ancestral state (supplementary fig. S3, Supplementary Material online), with the hugely accelerated appearance of a distinct vestibular ectoderm territory being the most prominent difference (Haag and Raff 1998; Love and Raff 2006; Koop et al. 2017; McDonald et al. 2024; Fig. 1c). Other notable differences include the apparent absence of endodermally derived neurons and primary germ cells in *H. erythrogramma*.

### scRNA-seq Data Accurately Reflects Known Regulatory Interactions in *L. variegatus*

The results presented above indicate that cells in the embryo of *H. erythrogramma* traverse rather different transcriptional trajectories (Figs. 3 and 4; supplementary fig. S4, Supplementary Material online) relative to *L. variegatus*, and that some differentiating cells emerge from distinct precursor populations in the two species (supplementary fig. S5, Supplementary Material online). These observations hint at evolutionary changes in underlying regulatory interactions. Two previous studies used scRNA-seq results to infer that specific regulatory interactions present in the sea urchin *Strongylocentrotus purpuratus* are absent in the sea star *Patiria miniata* (Foster et al. 2022; Spurrell et al. 2023). We built on this approach, defining criteria for inferring four distinct evolutionary scenarios: conserved interaction, conserved interaction but with a timing or spatial shift, novel interaction, and loss of interaction (supplementary fig. S6, Supplementary Material online).

**Fig. 5.**
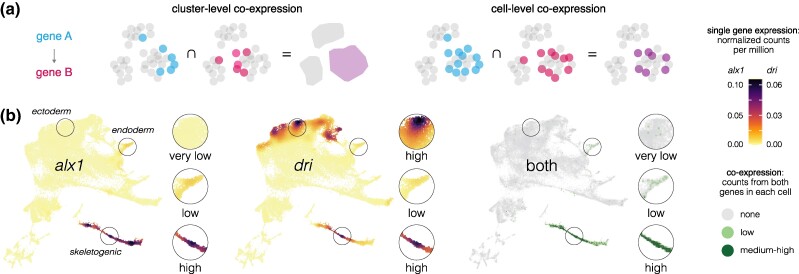
Measuring transcript co-expression. a) If gene A encodes a transcription factor that regulates the expression of gene B, both must be transcribed in the same cell (with some rare exceptions). *Cluster-level co-expression* measures whether both genes are expressed within the same cell cluster (pseudobulk). However, cluster-level co-expression can occur even if the two genes are never expressed in the same cells. This can happen when a cluster contains cells with diverging transcriptional states, a situation that arises during every cell fate specification event throughout development. *Cell-level co-expression* is a more stringent criterion that requires transcripts from both genes A and B to be present in the same cell, and is the definition applied in the present study. b) Example of an experimentally validated regulatory interaction that is reflected in the distribution of cell-level co-expression. *alx1* encodes a transcription factor that activates transcription of *dri* within the skeletogenic cell lineage (Oliveri et al. 2008). Co-expression of *alx1* and *dri* is detectable but low or very low and in a minority of cells in endoderm and ectoderm. Circles indicate regions shown at 2× magnification to the right of each UMAP. Robust and nearly universal co-expression occurs only within the skeletogenic cell lineage, precisely where it is predicted to occur. These results are consistent with an experimentally tested interaction and further imply that *alx1* does not influence *dri* expression outside the skeletogenic cell lineage. In this and all subsequent co-expression UMAPs, light dots represent very low co-expression (only 1 read detected from either or both genes), and dark dots represent moderate to high co-expression (at least 2 reads detected from both genes).

We analyzed co-expression of specific pairs of transcriptional regulators and their targets on a cell-by-cell basis to assess how accurately the presence of transcripts from both genes reflects experimentally validated regulatory interactions. Figure 5 explains how we measured cell-level co-expression and how this differs from cluster-level co-expression (pseudobulk). Note that cell-level co-expression as applied here is a more stringent measure than cluster-level co-expression, because it requires transcripts from both genes to be detected within the same cell and because it does not depend on how individual cells are assigned to clusters. Cell-level co-expression is also inherently conservative, as some cells expressing both genes are likely not counted due to transcript dropout resulting from the sparse nature of scRNA-seq data.

**Fig. 6.**
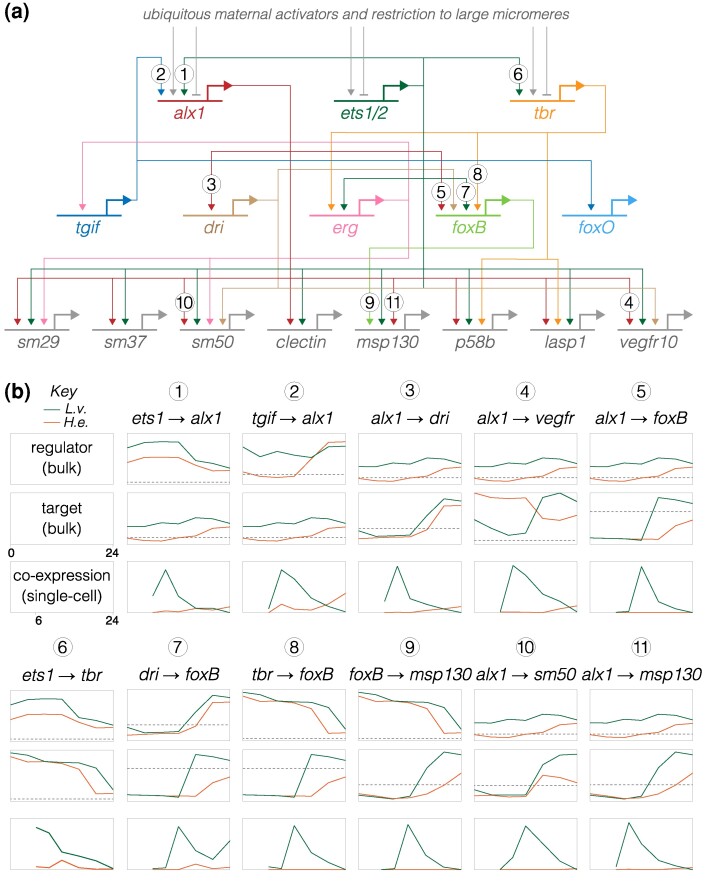
Inference of evolutionary changes in regulatory interactions based on proportion of co-expressing cells. a) Simplified version of the skeletogenic portion of the ancestral dGRN present in Camarodonta sea urchins with feeding larvae (adapted from Figs. 6 and 7 of Rafiq et al. (2012); built on data from Kurokawa et al. (1999), Davidson et al. (2002a), Oliveri et al. (2002, 2008), Ettensohn et al. (2003), Oliveri and Davidson (2004), Sharma and Ettensohn (2010), Rafiq et al. (2012)). The three primary activators of skeletogenic-specific transcription (top) feed directly or indirectly into a large set of effector genes, some of which are illustrated (bottom). b) Co-expression analysis of gene pairs involved in 11 experimentally validated regulatory interactions in the ancestral state (McDonald et al. 2024), represented by *L. variegatus* and compared with expression in *H. erythrogramma*. Numbers correspond to interactions in (a). The top two plots for each interaction show expression of regulator and target based on bulk RNA-seq (Israel et al. 2016), with a log_2_  *y* axis. The dashed line indicates very low expression (an average of 5 counts per million reads across time points, averaged across 3 biological replicates), which is effectively the lower limit of reliable detection (Israel et al. 2016). Supplementary fig. S11, Supplementary Material online shows larger plots with values. The plot directly below shows the proportion of cells that co-express both genes based on scRNA-seq, with a linear *y* axis; these time points begin at 6 hpf, the first time point common to both data sets. Note that *y* axes are *not* equivalently scaled because genes have a wide range of expression and co-expression levels. Most gene pairs show a strong peak of co-expression at 9 hpf in *L. variegatus*, which then drops as skeletogenic cells stop dividing while other cell lineages continue to proliferate. In contrast, this peak is notably absent in *H. erythrogramma*; instead, co-expression is initially zero or very low at 9 hpf and rises modestly 16 to 24 hpf. These results point to a general delay in co-expression of regulatory and target in *H. erythrogramma* relative to *L. variegatus*.

As positive controls, we first examined experimentally validated regulatory interactions in *L. variegatus*, focusing on the well-studied skeletogenic cell lineage (Kurokawa et al. 1999; Davidson et al. 2002a, 2002b; Oliveri et al. 2002, 2008; Ettensohn et al. 2003; Oliveri and Davidson 2004; Sharma and Ettensohn 2010; Rafiq et al. 2012, 2014). Figure 6a shows a simplified version of the skeletogenic cell portion of the ancestral dGRN (based on Rafiq et al. 2012). Across the top are the three primary activators of skeletogenic cell-specific transcription, and across the bottom a few of the many known effector genes of differentiated skeletogenic cells; between them lie some of the transcription factors that reinforce the differentiated state. Many of these interactions have been confirmed in multiple studies and some in multiple species with the ancestral developmental mode (reviewed in McClay 2011).

**Fig. 7.**
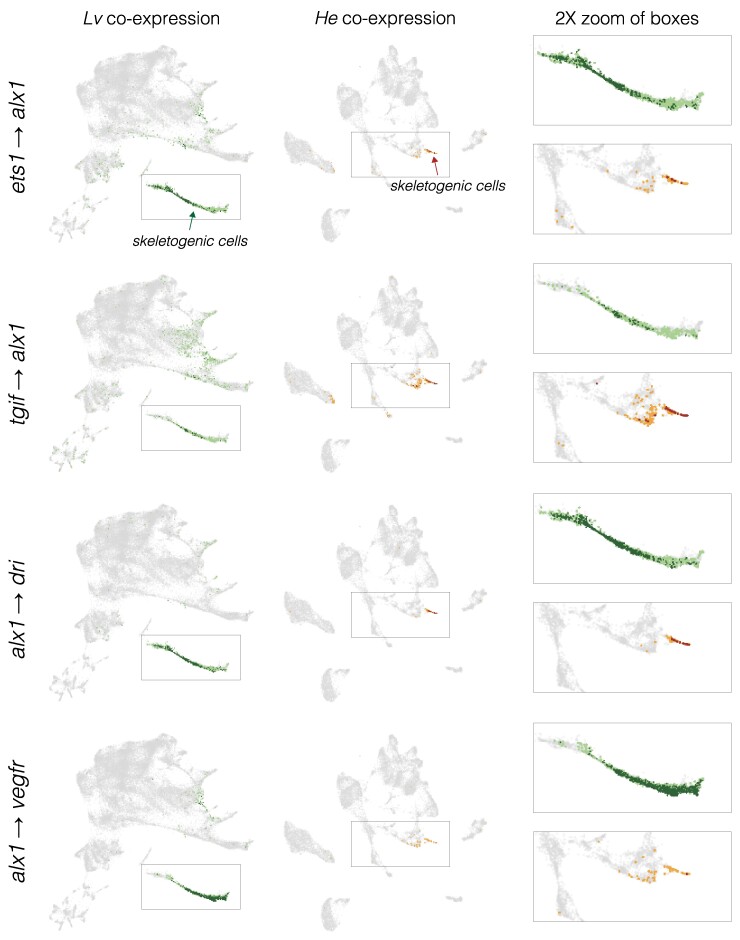
Inference of evolution changes in regulatory interactions based on the distribution of co-expressing cells. Co-expression analysis of experimentally validated regulatory interactions. UMAPs show the location of cells with co-expression of indicated regulator and target. *L. variegatus* = green dots and *H. erythrogramma* = orange dots; dark colors indicate cells with >2 UMIs for both regulator and target gene; pale colors indicate low co-expressing cells, where one or both genes have 1 or 2 UMIs. Boxes indicate areas shown at 2× in the right-hand column and arrows indicate skeletogenic cells in *H. erythrogramma*. See supplementary fig. S6, Supplementary Material online for a visual guide to interpreting evolutionary differences.

We initially focused on *alx1*, which encodes the master regulator of skeletogenic cell specification (Ettensohn et al. 2003; Sharma and Ettensohn 2010; Rafiq et al. 2012), examining interactions involving the two known activators of its transcription (*ets1* and *tgif*) and some of its many known targets (e.g. *dri*, *vegfr*, *sm50*, and *msp130*). We assessed co-expression in two ways: as the proportion of cells with co-expression over developmental time (Fig. 6b) and by the location of cells with co-expression within the first two dimensions of UMAP space (Fig. 7; supplementary fig. S7, Supplementary Material online).

Two general points stand out from the *L. variegatus* data (green lines and dots in Figs. 6b and 7; supplementary fig. S7, Supplementary Material online). First, co-expression of regulator and target occurs at the expected developmental stages. For the six *alx1* interactions shown in Fig. 6b, the proportion of cells expressing both regulator and target rises rapidly between 6 and 9 hpf, then declines over time as the skeletogenic cell precursors stop dividing while most other cell lineages continue to proliferate (Martik and McClay 2017). Note that the peaks of co-expressing cells are not evident in the bulk expression of the respective genes (plots immediately above). Many targets of *alx1* show delayed co-expression, with some not yet co-expressed at 6 hpf (e.g. *alx1*-*foxB*) or peaking after 9 hpf (e.g. *alx1*-*sm50*) (Figs. 6b and 7; supplementary fig. S7, Supplementary Material online). The delay in onset of structural gene expression is consistent with the gap of many hours between skeletogenic cell fate specification and differentiation (Rafiq et al. 2012, 2014).

Second, most co-expressing cells are restricted to the skeletogenic cell lineage and most cells in the skeletogenic lineage express both genes (Fig. 7; supplementary fig. S7, Supplementary Material online). This indicates that co-expression is readily detected despite the sparseness of scRNA-seq data. Both restriction to skeletogenic cells and presence in the vast majority of skeletogenic cells are consistent across many activator—target gene pairs involving *alx1* (Figs. 6b and 7; supplementary fig. S7, Supplementary Material online) as well as interactions involving other transcription factors within skeletogenic cells (supplementary fig. S8, Supplementary Material online). When a transcriptional activator is broadly expressed, co-expression with a given target gene typically involves a specific subset of the cells within its overall expression domain. For instance, *ets1* and *tbr* are expressed in the endomesoderm as well as in the skeletogenic cell lineage, but co-expression of *ets1-sm32* and *tbr-foxB* is limited to skeletogenic cells (supplementary fig. S9, Supplementary Material online). Other examples include *alx1-dri*, *otx-endo16*, *gataE-pks1*, and *alx1-sm30* (Fig. 5, supplementary fig. S9, Supplementary Material online).

**Fig. 8.**
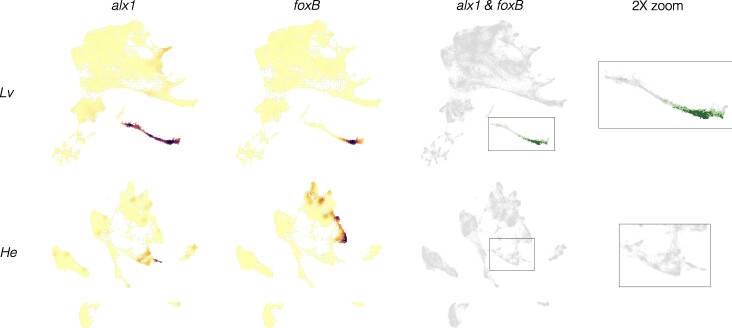
Inference of evolutionary loss of a regulatory interaction. a) Density plots showing expression of regulator (*alx1*) and target (*foxB*) genes in both species. Note that both *alx1* and *foxB* transcripts are readily detected in both species. b) Co-expression plots. The complete absence of co-expression in *H. erythrogramma* suggests that the ancestral *alx1*-*foxB* regulatory interaction has been lost in this species. See supplementary fig. S6, Supplementary Material online for a visual guide to interpreting evolutionary differences.

In some cases, cells outside the known area of interaction also co-express the regulator and target. Examples include *alx1-dri*, *ets1-alx1*, and *alx1-vegfr* (Figs. 6 and 8). In every such case examined, co-expression outside the known area of interaction comprised a substantially smaller proportion of cells and was dominated by cells with very low co-expression (light color). These cases of low levels of co-expression outside the area of the known interaction may indicate uncharacterized additional locations where the regulatory interaction actually occurs, or it may simply reflect noisy transcription with little or no functional consequence.

Overall, results are consistent with the developmental times and restriction to the skeletogenic cell lineage for these specific regulatory interactions in *L. variegatus*. Supplementary fig. S10, Supplementary Material online, shows a sampling of co-expression related to regulatory interactions in other embryonic territories. These are also largely consistent with the expected times and locations of experimentally validated regulatory interactions within the ancestral life history. For example, expression of *otx* is quite broad in the embryo but shows distinct patterns of co-expression with two different experimentally validated targets: in the endoderm, nonskeletogenic mesenchyme, and blastocoelar cells for *gataE*, but just in the endoderm for *endo16* (supplementary fig. S10, Supplementary Material online).

### A Subset of Regulatory Interactions may be Altered in *H. erythrogramma*

Next, we examined the same regulatory interactions in an evolutionary context (Figs. 7 and 8; supplementary figs. S7, S8, and S10, Supplementary Material online; *L. variegatus* = green and *H. erythrogramma* = orange). In most cases, co-expression occurs in the same cell lineage in both species. For instance, the ancestral interactions involving *alx1* are reflected as co-expression primarily within skeletogenic cells in *H. erythrogramma*. Similarly, *gataE* and its target *pks1* are co-expressed primarily in the pigment cells (supplementary fig. S9, Supplementary Material online). These results are consistent with conservation of ancestral regulatory interactions in *H. erythrogramma*. Several differences between species, however, point to evolutionary changes in specific regulatory interactions, including changes in timing (earlier or later), location (extent or cell lineage), and presence/absence (supplementary fig. S6, Supplementary Material online).

Differences in timing of co-expression are common. Several examples are evident in Fig. 6b: the prominent early peak of *alx1* interactions in *L. variegatus* is reduced or entirely absent in *H. erythrogramma*; instead, the proportion of co-expressing cells rises later in *H. erythrogramma*, reflecting much later differentiation. This is largely due to a late rise in *alx1* expression (Fig. 6b; dashed line indicates <5 transcripts per million across 3 replicates). Among targets of *alx1* expression, *dri* shows a similar expression profile among species, while *vegfr* shows highly divergent expression; nonetheless, the co-expression time courses and UMAPs are very similar for both interactions. The simplest explanation for these results is that some regulatory interactions take place in *H. erythrogramma* but that they are considerably delayed relative to *L. variegatus*. Earlier observations indicating a delay in both specification and differentiation of skeletogenic cells in *H. erythrogramma* (Figs. 1, 3, and 4; supplementary fig. S5, Supplementary Material online) are consistent with this interpretation. Co-expression of several other experimentally validated interactors outside the skeletogenic lineage also shows temporal differences (supplementary fig. S10, Supplementary Material online). Although we did not attempt a systematic assessment throughout the dGRN, evolutionary differences in the timing of co-expression appear to be widespread.

Less commonly, an interaction appears to be absent in *H. erythrogramma*. For the ancestral interaction *alx1-foxB*, no cells at any time contain reads from both genes in *H. erythrogramma* (Fig. 8). Since both genes are robustly expressed at other times and locations in the *H. erythrogramma* embryo, the complete absence of co-expression is probably not a technical issue with detection. Another example is the ancestral interaction *alx1*-*foxO*, where in *H. erythrogramma* only one cell across all time points expresses both genes and it contains low UMI counts from each gene (indicated by light orange; supplementary fig. S7, Supplementary Material online). Given that co-expression often occurs in a small number of scattered cells outside the region where a specific interaction is thought to occur (Fig. 8; supplementary figs. S7, S8, and S10, Supplementary Material online), this low level of co-expression of *alx1* and *foxO* in *H. erythrogramma* is likely not functionally significant. Other examples involve *tbr*, which is not expressed in skeletogenic cells in *H. erythrogramma*, despite being expressed elsewhere in the embryo (supplementary fig. S8, Supplementary Material online). The simplest interpretation is that these regulatory interactions do not take place in *H. erythrogramma*. We found far fewer cases of likely loss of a regulatory interaction than a change in timing, although we did not attempt a formal quantitative comparison due to the small number of cases examined relative to the entire transcriptome.

Although the focus here has been on skeletogenic cells, the same general findings are evident in other territories (supplementary fig. S10, Supplementary Material online). Again, most co-expression of regulator and target in *L. variegatus* corresponds to expected times and locations. When comparing species, most co-expression occurs in the same embryonic territory or cell lineage, but with notable exceptions that suggest specific kinds of evolutionary change (supplementary fig. S6, Supplementary Material online). Among these, differences in timing or location are common. For instance, co-expression of *otx* and *gataE* occurs throughout the endomesoderm of both species, but is largely confined to 9 to 12 hpf in *H. erythrogramma* while present from 6 to 24 hpf in *L. variegatus*. Similarly, co-expression of *bra* and *foxA* is largely endodermal in both species, but in addition is more extensive in the ectoderm of *H. erythrogramma* than in *L. variegatus*. A few additional likely losses of regulatory interactions are also evident. These include *bra*-*apobec* within the endoderm, which may be absent in *H. erythrogramma* (supplementary fig. S10, Supplementary Material online). Due to the large number of documented interactions within the ancestral dGRN, a comprehensive co-expression analysis throughout the dGRN is beyond the scope of the present study. In addition, we did not attempt to examine regulatory interactions outside the dGRN, so the general trends evident in our results may not apply more broadly (for instance, to the evolution of regulatory interactions involving genes encoding metabolic enzymes or cytoskeletal proteins).

## Discussion

Comparisons of single-cell transcriptomes between species have been used to document the presence or absence of cell types (e.g. Cary et al. 2020; Levy et al. 2021; Tarashansky et al. 2021; Wang et al. 2021; Woych et al. 2022; Alvarez-Campos et al. 2024; Mah and Dunn 2024), but less commonly to understand how developmental mechanisms evolve and contribute to organismal traits. This study used scRNA-seq to examine the evolution of cell fate specification and differentiation in *H. erythrogramma*, a sea urchin with a recently modified life history (Fig. 1a; Raff 1992; Zigler et al. 2003; Wray 2022). The goal was to gain insights into the developmental basis for massively modified larval morphology and hugely abbreviated premetamorphic development. We generated a developmental time course of scRNA-seq data from *H. erythrogramma* and carried out comparative analyses with our published data for *L. variegatus* (Massri et al. 2021), representing the ancestral life history in sea urchins (McEdward and Miner 2001; Raff and Byrne 2006). This discussion is organized around three broad themes revealed by comparative analyses of the scRNA-seq time courses.

### Evolution of Embryonic Patterning

The earliest indication that embryonic patterning might be modified in *H. erythrogramma* came from observations of cleavage divisions, which differ from the stereotypical pattern in the ancestral life history. In sea urchins with feeding larvae, unequal vegetal cleavage divisions establish the clonal founders of two distinct cell lineages: the germ line (Pehrson and Cohen 1986; Oulhen et al. 2019) and the skeletogenic cells, which also become the primary signaling center of the embryo (Hörstadius 1973; Sherwood and McClay 1999; Sweet et al. 2002; Wikramanayake et al. 2004; Wei et al. 2012). These processes appear to be conserved in *H. tuberculata*, a close relative of *H. erythrogramma* (Fig. 1a; Wray and Raff 1989; Love et al. 2008; Morris et al. 2019). Over the next few hours, a series of inductive interactions initiated by the primary signaling center specify other embryonic cell lineages (reviewed in McClay 2011). These critical early patterning events are broadly among sea urchin species with the ancestral life history (McClay 2011; Thompson et al. 2015; Minokawa 2017; Yamazaki et al. 2021; Fig. 1a). In contrast, *H. erythrogramma* lacks any early unequal cleavage divisions (Williams and Anderson 1975). Dye-tracer studies reveal a general delay in specification and, specifically, that no early blastomeres are clonal founders of either the germ line or skeletogenic cells (Wray and Raff 1989, 1990).

The scRNA-seq results reported here confirm this delay and add new information. At 6 hpf, the embryo of *L. variegatus* contains four transcriptionally distinct populations of cells: germ cell precursors, skeletogenic cell precursors, early ectoderm, and endomesoderm (Massri et al. 2021; Fig. 1b). In contrast, the 6 hpf embryo of *H. erythrogramma* contains a single population of cells producing transcripts characteristic of undifferentiated epithelium (Fig. 1c;  supplementary fig. S2, Supplementary Material online).

Closer examination of each of the three early patterning events reveals striking changes in early patterning. (i) Skeletogenic cells: Both species express *alx1*, which encodes the master regulator of skeletogenic cell fate (Ettensohn et al. 2003), but with a large delay from ∼3 hpf in *L. variegatus* to later than 12 hpf in *H. erythrogramma* (Fig. 1c; supplementary figs. S2 and S7, Supplementary Material online). A distinct skeletogenic transcriptional state is apparent by ∼6 hpf in *L. variegatus* but not until ∼16 hpf in *H. erythrogramma*. Even at 30 hpf, skeletogenic cells of *H. erythrogramma* are not as differentiated as they are at 24 hpf in *L. variegatus* (Fig. 4). (ii) Germ cells: At no point up to 30 hpf in *H. erythrogramma* is there a distinct cell population expressing germ cell markers. Species with the ancestral life history express *nanos2* broadly, but transcripts and protein accumulate exclusively within the small micromeres (Oulhen et al. 2019). Expression of *nanos2* also occurs in *H. erythrogramma*, but does not become localized, remaining widespread in endomesoderm up to 30 hpf (supplementary fig. S2, Supplementary Material online). The same is true of *vasa*, another germ line marker (supplementary fig. S2, Supplementary Material online). (iii) Primary signaling center: In *L. variegatus*, the skeletogenic precursors between 3 and 6 hpf express genes that encode signaling ligands, including *wnt1*, *wnt8*, and *delta* (Massri et al. 2021). In *H. erythrogramma*, however, these genes are co-expressed with markers of endomesoderm (*foxA*, *ism*, *blimp1*; supplementary fig. S2, Supplementary Material online) and never with skeletogenic markers, suggesting that the primary signaling center is spatially separated from skeletogenic cell fate specification. In addition, the timing of expression differs in *H. erythrogramma*: only *wnt8* is expressed at 6 hpf, while all three transcripts show peak expression at 9 hpf (supplementary fig. S2, Supplementary Material online). These observations indicate that the three earliest embryonic patterning events in the ancestral state are all delayed in *H. erythrogramma*, and that they have become spatially and temporally separated from each other. This delay is reflected more broadly in the embryo, with transcriptional states in multiple territories diverging later on average (Figs. 3 and 4; supplementary fig. S4, Supplementary Material online).

The simplest model to explain these observations is that embryonic patterning mechanisms are conserved in *H. erythrogramma* but activated later in development. Three lines of evidence suggest that the situation may actually be more complicated. First, the three earliest patterning events are nearly simultaneous in the ancestral state but occur at widely separated times in *H. erythrogramma*: the primary signaling center is established prior to 9 hpf, skeletogenic cell fate specification takes place between 12 and 16 hpf, and germ cell fate specification occurs sometime after 30 hpf. Second, in a previous study, we showed through perturbation experiments that the earliest regulatory interactions responsible for skeletogenic cell fate specification have been lost in *H. erythrogramma* (Davidson et al. 2022a). Germ cell fate specification has not been experimentally investigated in *H. erythrogramma*, but *foxY*, which encodes a key regulator of *nanos2* transcription in the ancestral life history (Oulhen et al. 2019), is not tightly co-expressed with it. Third, some populations of larval cell types in *H. erythrogramma* derive from different founder cells than in the ancestral condition (supplementary fig. S5, Supplementary Material online). In particular, pigment cells and blastocoelar (immune) cells derive from a uniquely shared population of nonskeletogenic mesenchyme cells in *L. variegatus* (Fig. 1b) and other species with the ancestral life history (McClay 2011); in contrast, in *H. erythrogramma*, these two cell types derive from spatially and temporally distinct source populations, and instead, it is blastocoelar and skeletogenic cells that share a common origin (Fig. 1c;  supplementary fig. S5, Supplementary Material online). Thus, embryonic patterning and cell fate specification appear to be rearranged in a manner inconsistent with a simple conservation-with-delay model.

Importantly, not all embryonic patterning events are delayed in *H. erythrogramma*. A striking counter example is the breaking of left–right symmetry, which occurs before first cleavage (Henry and Raff 1990; Henry et al. 1990). In contrast, the first indication of left–right asymmetry in the ancestral developmental mode occurs in the late gastrula (Duboc et al. 2005; Bessodes et al. 2012). Another accelerated patterning event in *H. erythrogramma* involves the early establishment of the imaginal adult rudiment, which begins at about 30 hpf in *H. erythrogramma* (Williams and Anderson 1975; Wray and Raff 1989; Koop et al. 2017) but not until several days postfertilization in the ancestral condition (Lowe et al. 2002; Formery et al. 2022).

In sum, patterning mechanisms in the early embryo of *H. erythrogramma* appear to represent a complex mosaic of changes. Three critical early patterning events that are tightly associated with a set of unequal cleavages in the very early embryo of the ancestral state are delayed in *H. erythrogramma*, and in addition are dissociated from each other in time and location. In contrast, some other pivotal patterning events are accelerated in *H. erythrogramma*. Furthermore, the origins of some larval cell types have been rearranged, likely reflecting changes in embryonic cell lineages. Together, these changes suggest that several modifications have evolved in interactions within the dGRN, as discussed next.

### Evolution of Regulatory Interactions During Development

The ability to assay transcription from single cells provides exciting opportunities to investigate the evolution of transcriptional regulation. In particular, the interaction between a transcriptional activator and a regulatory target should be reflected by co-expression within the same cell. It is important to emphasize that co-expression does not by itself constitute direct evidence: it can reveal a pattern consistent with a regulatory interaction, but experimental evidence is needed to confirm. For this reason, we restrict attention here to gene pairs representing experimentally documented regulatory interactions in the ancestral state, rather than attempting to identify previously unknown interactions. Our current understanding of ancestral dGRN interactions in sea urchins comes primarily from three species: *S. purpuratus*, *L. variegatus*, and *Paracentrotus lividus*, all of which have the ancestral life history and diverged ∼35 to 50 Ma (Fig. 1a). Most regulatory interactions that have been experimentally tested in multiple species appear well conserved, as are expression timing and domains of most of genes (McClay 2011; Gildor and Ben-Tabou De-leon 2015; Israel et al. 2016; Massri et al. 2023).

We first assessed how well scRNA-seq captures previously documented regulatory interactions in *L. variegatus* by analyzing the distribution of regulator and target gene co-expression during development (Figs. 6 and 7; supplementary figs. S7, S8, and S10, Supplementary Material online). In each case examined, co-expression corresponds to known developmental times and locations of specific regulatory interactions. For instance, *ets1* and *tbr* are expressed throughout the endomesoderm, but *ets1-sm32* and *tbr-foxB* are co-expressed exclusively within the skeletogenic cell lineage and only beginning at ∼12 hpf (supplementary fig. S9, Supplementary Material online). Co-expression is readily detected for all gene pairs, despite the sparseness of scRNA-seq data. For most interactions, most cells in the expected territory express multiple transcripts: dark green dots in the UMAPs indicate individual cells containing at least 2 UMIs from each gene, while light green indicates just 1 UMI for one or both genes. Overall, co-expression plots are consistent with results from prior experimental studies and are sufficiently sensitive that absence of co-expression is biologically meaningful.

Based on this information, it is possible to make inferences about evolutionary conservation and change by examining co-expression of gene pairs among species (see supplementary fig. S6, Supplementary Material online, for an explanation of how evolutionary inferences are called). Comparisons of co-expression are shown in Figs. 6 and 7, and supplementary figs. S7, S8, and S10, Supplementary Material online (*L. variegatus* = green, *H. erythrogramma* = orange). (i) Conservation of an interaction: Most gene pairs show co-expression in the same embryonic territories or cell types in both species. Co-expression in a completely distinct location from *L. variegatus* was not observed in *H. erythrogramma* for any of the gene pairs examined. The most straightforward interpretation of this pattern is that the ancestral regulatory interaction occurs during development in *H. erythrogramma*. (ii) Temporal and/or spatial shift in a conserved interaction: Although the location of co-expression was largely conserved in *H. erythrogramma*, its timing and extent typically were not. Most shifts in timing involved a delay in the appearance of co-expression in *H. erythrogramma* relative to *L. variegatus*. Among many examples are *ets1-alx1*, *tgif-alx1*, *alx1-dri*, *alx1-vegfr*, and *ets1-delta* (Figs. 6b and 7; supplementary figs. S7 and S8, Supplementary Material online). These cases are consistent with the general delay in specification and differentiation in *H. erythrogramma* shown in Figs. 2–4, and supplementary fig. S4, Supplementary Material online. While the timing of developmental gene expression can differ among sea urchin species with the ancestral life history, those shifts are typically smaller in magnitude and not biased in direction (Gildor and Ben-Tabou de-Leon 2015; Israel et al. 2016; Massri et al. 2023). Clear examples of evolutionary differences in the extent of co-expression include all interactions specific to the gut and skeletogenic cells, both of which involve proportionally far fewer cells in *H. erythrogramma* (Fig. 8; supplementary figs. S7, S8, and S10, Supplementary Material online). (iii) Loss of an interaction: A minority of gene pairs that are co-expressed in the expected location in *L. variegatus* show no or barely detectable co-expression in *H. erythrogramma*. Examples include *alx1-foxB*, *tbr-foxB*, *tbr-lasp1*, and *bra-apobec* (Figs. 7b and 8; supplementary figs. S7, S8, and S10, Supplementary Material online). In these and other cases, lack of co-expression is not due to a technical issue with detection, as transcripts from both genes are detected elsewhere in the embryo (supplementary fig. S2, Supplementary Material online). The most straightforward interpretation is that the specific regulatory interaction likely does not occur in *H. erythrogramma*. These cases are prime candidates for experimental validation through knockdowns of the regulator.

Inferred evolutionary changes in regulatory interactions in *H. erythrogramma* are not randomly distributed across the developmental gene regulatory network, but instead concentrated around particular developmental processes. As discussed earlier, a very early patterning event in the ancestral dGRN is the establishment of cells that are both the founders of the skeletogenic cell lineage and the primary signaling center. In *L. variegatus*, genes encoding the key transcriptional activators of the skeletogenic cell lineage, *alx1* and *ets1*, are first expressed at about the same time as genes encoding signaling ligands (*wnt1*, *wnt8*, and *delta*; Massri et al. 2021). In contrast, expression of these genes occurs in two distinct phases and locations in *H. erythrogramma*: an earlier phase in the archenteron involving genes that encode ligands (peaking at 9 hpf and greatly reduced by 12 hpf), and a later phase in the mesenchyme involving genes specific to skeletogenic cells (begins ∼16 hpf; Fig. 7b; and supplementary fig. S2, Supplementary Material online). These results suggest that two key patterning events that are co-localized in the ancestral state have become independently regulated during the origin of the derived life history. This is remarkable, given the prior conservation of the ancestral state for over 230 My (Thompson et al. 2015; Erkenbrack et al. 2018; Yamazaki et al. 2021).

The most obvious way a regulatory interaction could be lost during evolution is whether the regulator is simply not expressed in the appropriate cell lineage or territory within the embryo. This is the case for two transcription factors, *tbr* and *foxB*. Both are expressed within the skeletogenic cell lineage of *L. variegatus* (Saunders and McClay 2014), but the scRNA-seq data from *H. erythrogramma* do not reveal any expression within these cells despite clear expression elsewhere in the embryo (supplementary fig. S2, Supplementary Material online). Indeed, of the 11 genes known to encode transcription factors that activate expression within the skeletogenic cell lineage of species with the ancestral life history (Oliveri et al. 2008; Saunders and McClay 2014; Rafiq et al. 2012), *tbr* and *foxB* are only 2 that are not expressed in these cells in *H. erythrogramma*. The absence of *tbr* expression may have limited impact on the expression of effector genes in skeletogenic cells, as *tbr* appears to have far fewer targets than *alx1* and *ets1*, the two primary activators of skeletogenic-specific transcription (Rafiq et al. 2012). The four known effector gene targets of *tbr* are all expressed in skeletogenic cells of *H. erythrogramma*, likely because they also receive input from other transcriptional activators, including *alx1* and *ets1* (Rafiq et al. 2012). *tbr* was previously proposed to be a more recent evolutionary addition to the skeletogenic cell GRN due to having fewer regulatory targets than *alx1* and *ets1* (Rafiq et al. 2012). The evolutionary loss of *tbr* expression within skeletogenic cells may have been possible for the same reason, coupled with the fact that nine other genes encoding transcription factors with roles in activating effector gene expression are also expressed within skeletogenic cells, thus providing some degree of regulatory redundancy.

### Evolution of Morphology and Life History

The evolution of massive maternal provisioning in *H. erythrogramma* also precipitated changes in larval morphology and life history traits (Raff and Byrne 2006; Wray 2022). The most obvious are loss of feeding structures and a functional digestive tract (Williams and Anderson 1975), which are no longer needed with a richly provisioned egg (Hoegh-Guldberg and Emlet 1997; Byrne et al. 1999; Davidson et al. 2019). Another set of changes was likely driven by selection to reduce larval mortality by shortening premetamorphic development, including earlier left–right symmetry breaking, differentiation of coeloms, and formation of the adult imaginal rudiment (Williams and Anderson 1975; Wray and Raff 1989; Henry et al. 1990; Koop et al. 2017).

The scRNA-seq data reflect both sets of changes in the proportions of cell types in the early larva (Fig. 1d). In *H. erythrogramma*, far fewer cells are allocated to endoderm, which is nonfunctional until after metamorphosis, and to skeletogenic cells, which produce a vestigial larval skeleton (Williams and Anderson 1975; Emlet 1995). Conversely, more cells are allocated to coeloms and ectoderm, both of which contribute substantially to accelerated development of the post-metamorphic juvenile (Williams and Anderson 1975; Wray and Raff 1989; Koop et al. 2017).

In sea urchins with the ancestral life history involving feeding larvae, four territories of ectodermal cells are evident from anatomy and gene expression: a ciliated band used for feeding and locomotion, an anterior neurogenic domain, and generalized ectoderm with distinct oral and aboral domains. These territories are recovered as separate clusters with scRNA-seq in *L. variegatus* (Fig. 1b;  supplementary fig. S3, Supplementary Material online). Previous studies examining ectodermal gene expression in *H. erythrogramma* found no evidence of conserved oral and aboral territories, and suggested instead that the ancestral ectodermal domains are reorganized (Haag and Raff 1998; Love and Raff 2006; Koop et al. 2017). The scRNA-seq data reveal a well-defined anterior neurogenic domain in *H. erythrogramma* (supplementary figs. S2 and S3, Supplementary Material online). However, the other ancestral ectodermal domains are more difficult to recognize in the *H. erythrogramma* larva. Markers of oral and aboral ectoderm the ancestral state are not consistently co-localized in *H. erythrogramma* (supplementary fig. S3, Supplementary Material online). The ciliated band, which is used for feeding, has been lost in *H. erythrogramma* (Williams and Anderson 1975). The only regions of dense cilia in the *H. erythrogramma* larva likely correspond instead to the epaulettes of late larvae in the ancestral state (Byrne et al. 2001), which are used exclusively for swimming (Emlet 1995). The other derived trait in *H. erythrogramma* that likely contributes to changes in expression of regulatory genes within the ectoderm is the greatly accelerated development of the imaginal adult rudiment (Williams and Anderson 1975; Wray and Raff 1989; Emlet 1995; Koop et al. 2017). Vestibular ectoderm is a distinct gene expression territory within the ectoderm of *H. erythrogramma* by 24 hpf (Koop et al. 2017). Comparative analysis of gene expression in the epaulettes and vestibule will require extending the *L. variegatus* scRNA-seq time course to late larval stages, as these structures have not yet developed in the early larva.

## Conclusion

The scRNA-seq data presented here reveal numerous features of development in *H. erythrogramma* that are likely conserved and others that are likely modified since its divergence from other sea urchins that share the ancestral life history. While scRNA-seq data alone do not provide direct evidence about molecular mechanisms, they can produce detailed information about specific developmental processes that are not evident from bulk RNA-seq and would otherwise require gene-by-gene expression analyses. Here, we report a close correspondence between evolutionary changes in the timing and location of regulatory gene expression and evolutionary changes in larval morphology and life history. Many specific regulatory interactions that are widely conserved among sea urchins with the ancestral life history appear to be conserved but delayed in *H. erythrogramma*, while a small number may have been lost entirely. These results provide specific predictions that can be tested efficiently using perturbation experiments, greatly facilitating the daunting challenge of understanding which connections within developmental gene regulatory networks are conserved, altered, or lost during the course of evolution.

## Materials and Methods

### Spawning and Embryo Culture

Adult *H. erythrogramma* were collected under permit near Sydney, Australia, during October and November. Crosses were initially established for the purpose of optimizing dissociation protocols; subsequently, a single cross was used to source samples for this study. Adults were spawned by injecting 0.5 mL 0.5 M KCl intracoelomically. Unfertilized eggs were allowed to float and washed 3× in filtered natural sea water (FNSW). Eggs were fertilized with sperm in FNSW containing 0.02 g PABA/100 mL. Zygotes were washed an additional 3× in FNSW to remove residual sperm and PABA and embryos cultured at 23 °C in FNSW. At each time point, embryos were visually verified to be morphologically similar prior to dissociation. Throughout, methods closely matched our previous scRNA-seq analysis of *L. variegatus* (Massri et al. 2021), including only slightly species-optimized dissociation protocols, same rearing temperature, time-matched samples, and same versions of 10× library kits and Illumina sequencing chemistry.

### Time Points Sampled

Embryos/larvae were sampled at seven time points: 6, 9, 12, 16, 20, 24, and 30 hpf (late cleavage through early larva). Time points were chosen to align with Massri et al. 2021, with two additional considerations. First, due to the large egg size of *H. erythrogramma* (∼430 µm diameter), blastomeres exceed the diameter of the microfluidics on the 10× platform until the 512-cell stage (6 hpf), which became our first time point. Second, prior studies suggested that activation of the zygotic genome in *H. erythrogramma* is somewhat delayed relative to *L. variegatus* (Wang et al. 2020; Davidson et al. 2022a, 2022b); thus, we collected one additional time point (30 hpf) beyond the last sampled in *L. variegatus* (24 hpf). Comparative analyses drew on published data from Massri et al. 2021 for *L. variegatus* and from the present study for *H. erythrogramma*.

### Cell Dissociation and Fixation

At each time point, the culture was subsampled and embryos washed two times in calcium-free artificial seawater (CFASW). Approximately 3 mL embryos in CFASW were added to 7 mL of dissociation buffer (1.0 M glycine and 0.25 mM ethylenediaminetetraacetic acid, pH 8.0 with HCl) at 4 °C, and then placed on a rocker for 4 min. Following incubation, samples were triturated 10 to 15×, then 10 mL of ice-cold methanol was added, and incubated for 4 additional minutes on a rocker. Following incubation, samples were triturated 10 to 15× additionally, and visually inspected under a microscope for a homogenous single-cell suspension. To fix cells, 40 mL of ice-cold methanol was added to a final concentration of 80%. Samples were then placed on a rocker for 1 h prior to storage at −20 °C.

### Library Preparation and Sequencing

Fixed cells were washed once in methanol, then rehydrated by washing in 3× sodium citrate buffer. Cell concentrations were determined using a hemacytometer. Seven libraries were prepared using the 10× Genomics 3′ v3 gene expression kit and the 10× chromium platform to encapsulate single cells within droplets. Library quality was verified using an Agilent 2100 Bioanalyzer. Libraries were titered and pooled at Duke University's Sequencing and Genomic Technologies Core Facility, then sequenced in one S1 flow cell on an Illumina NovaSeq 6000 with 28 × 8 × 91 bp.

### Initial Processing and Production of Raw CSV Count Files

Following sequencing, Cellranger 3.1.0 was used to convert Illumina-generated BCL files to fastq files using the Cellranger “mkfastq” command. The “mkref” command was then applied to index the *H. erythrogramma* 1.0 Genome (Davidson et al. 2022a). The “count” command was used to demultiplex and quantify reads mapping to the reference *H. erythrogramma* genome. The “mat2csv” command was used to generate CSV RNA count matrix files for each time point for downstream analysis.

### Data Filtering and Normalization

All 19 *L. variegatus* and 7 *H. erythrogramma* CSV RNA count matrix files were uploaded to R, and a merged Seurat object (Hao et al. 2024) was generated for each species. The *L. variegatus* Seurat object was filtered to remove lower quality cells with nFeature_RNA > 200, nFeature_RNA < 7,000, and nCount_RNA < 10,000. In total, 50,638 *L. variegatus* cells remained. The *H. erythrogramma* Seurat object was filtered with nFeature_RNA > 200, nFeature_RNA < 4,000, and nCount_RNA < 10,000. In total, 23,156 *H. erythrogramma* cells remained. SCTransform was then independently applied to the *L. variegatus* merged filtered object and the *H. erythrogramma* merged filtered object to perform normalization, and regression of ribosomal and cell cycle–related genes using the command: *vars.to.regress*  *=*  *c(”percent.Rb”, “cell.cycle”)*. These metacolumns were added with the following commands: *PercentageFeatureSet(merged, pattern*  *=*  “\\b\\w*Rp[sl]\\w*\\b”, col.name  *=*  *“percent.Rb”) and PercentageFeatureSet(merged, pattern*  *=*  “\\b\\w*C[d|y]c\\w*\\b”, col.name  *=*  *“cell.cycle”)*.

### Dimensionality Reduction, Visualization, and Clustering

We next independently performed principal component analysis on the SCTransformed *L. variegatus* Seurat object and *H. erythrogramma* Seurat object, and found the nearest neighbors and clusters (Hao et al. 2024). UMAP was then applied to each species to visualize the multidimensional scRNA-seq in a two-dimensional space. Each species cluster was annotated using co-expression of dGRN genes, and published in situ hybridization patterns as markers. Echinobase (Arshinoff et al. 2022) was used to identify gene function. See Massri et al. (2021) for a list of marker genes and supporting literature.

### Multispecies Integrated Analysis

Orthologroups were identified using OrthoFinder v 2.5.4 (Emms and Kelly 2019) and used to generate a list of 1:1 orthologs in *L. variegatus* and *H. erythrogramma*. In total, 7,349 of the genes expressed in the combined data set were identified as 1:1 orthologs. The standard Seurat/SCTransform pipeline was performed, and then integrated by species using the CCA workflow (Butler et al. 2018) using *L. variegatus* as the reference.

### Waddington OT Developmental Trajectories

To infer developmental trajectories in *H. erythrogramma*, we used Waddington OT (Schiebinger et al. 2019). To execute, we used the SCTransform normalized expression matrix obtained after running Seurat, a table of cell barcodes with cell-type annotations, and a growth rate table that was estimated from expected changes in lineage proportions over time using the model implemented in Waddington OT. To estimate cell division rates, we used the best estimate of the expected number of cells at key developmental time points. We assumed that cell divisions were uniform between estimates of expected cell numbers. Next, we recalculated transport maps using the modeled cell division rates, optimization parameters *ε* = 0.05, *λ*1 = 1, and *λ*2 = 50, and 20 iterations of growth rate learning. We used the transport map model throughout our analysis, which included triangle plots and lineage trees.

### Waddington OT Time Alignment

To estimate timing differences between the *L. variegatus* and *H. erythrogramma* data sets, we used optimal transport combined with the gene orthology tables. First, we used the previously calculated transport maps for both data sets to obtain fate probabilities for the cells at each time point. Fate probabilities were computed relative to cell types found in the last time point of their respective data set. Next, we restricted the normalized counts for *L. variegatus* and *H. erythrogramma* to known gene orthologs using the previously generated gene ortholog table. Then, for each cell type, a time point by time point matrix of earth mover distances between the two data sets was computed. In the calculations for each cell type and pair of times, cells were weighted by their fate probabilities to the cell type in question. Finally, for each *L. variegatus* time point, the *H. erythrogramma* time point corresponding to the minimum earth mover distance to it was found. These pairs were found for each cell type. We then take these pairs of time points to be the optimal developmental time alignments for the cell type.

### Waddington OT Triangle Plots

To construct triangle plots, we followed the approach used in our previous analysis of *L. variegatus* (Massri et al. 2021). Briefly, we used transport maps calculated above to compute fate probabilities with respect to the last common time point in our data set (24 hpf) and visualized them by computing the barycentric coordinates of cell fates between two different cell types and at a threshold of 0.7.

### Developmental Lineage Trees

To infer cell lineage trees, we used our modeled transport maps to find connections between cell clusters by calculating the fraction of descendants that end up in cluster/cell-type *j* at time *ti*  *+*  *1* from cluster/cell-type *i*. The minimum number of cells for a cluster to be represented set to 10, and the minimal edge weight cutoff was set to 0.15. Once the unwanted edges were removed, the data were written in a format that is usable by d3.js.

### Co-expression Analyses

We consider two genes, A and B, to be co-expressed if at least 1 read is mapped to each gene in a single cell. In other words, co-expression is the intersection of the set of cells expressing gene A and the set of cells expressing gene B. We calculated co-expression in two ways. The first generates a time course of the percentage of cells at each stage that express both gene A and gene B (Fig. 6b). We used a custom Python script to tally the number of cells containing at least one mapped read from gene A and from gene B within every cell at each time point directly from the count tables; these values were then normalized by the total number of cells for each sample cell, and grouped in time courses for both species. A custom R script was then used to visualize co-expression time courses. The second assigns color values to every cell in a UMAP plot as gray (no co-expression), light green or orange (low co-expression), or dark green (moderate to high co-expression). We define low co-expression as cells containing only 1 read from one or both genes A and B; moderate-to-high co-expression is thus any cell containing at least 2 reads from both genes. A separate custom R script was used to generate plots of co-expression in UMAP space (Figs. 7 and 8; supplementary figs. S7 to S10, Supplementary Material online).

## Supplementary Material

evae258_Supplementary_Data

## Data Availability

The data underlying this article are available in GEO at https://www.ncbi.nlm.nih.gov/geo/ and can be accessed with GSE277517. Code can be accessed at: https://github.com/Wray-Group-at-Duke/single-cell_coexpression.
