# Supplementary material for: Single-Cell Transcriptomics Reveals Evolutionary Reconfiguration of Embryonic Cell Fate Specification in the Sea Urchin Heliocidaris erythrogramma
Source: Genome Biol Evol. 2024 Nov 26;17(1):evae258. doi: 10.1093/gbe/evae258 (PMC11719709; doi:10.1093/gbe/evae258)
Supplement: evae258_Supplementary_Data [file evae258_supplementary_data.zip › Massri&al_Supp.docx]

SUPPLEMENTARY FIGURES AND TABLES


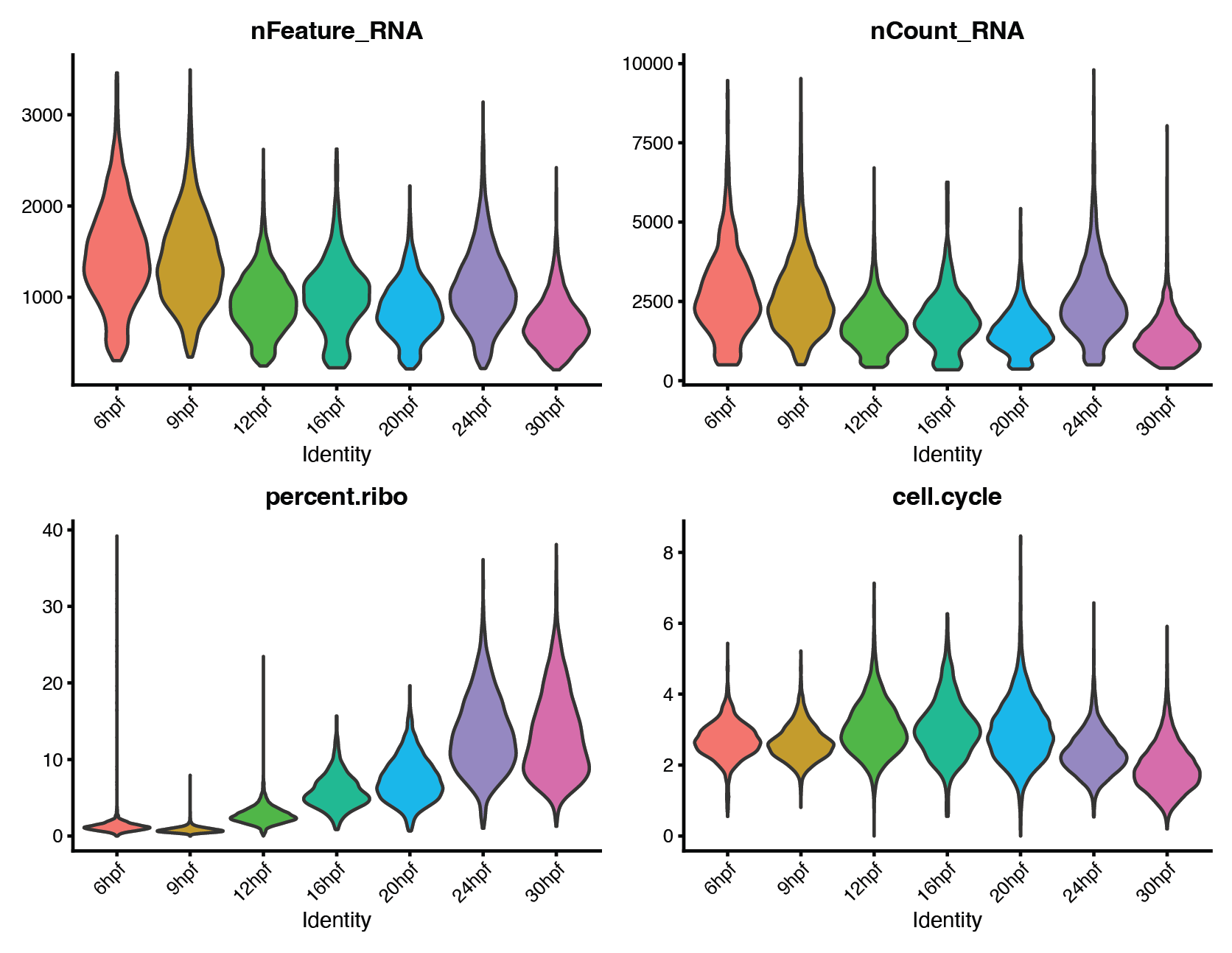


**Figure S1. Metrics of scRNA-seq transcriptomes over development in *H. erythrogramma*.** Violin plots showing the frequency distribution of four informative metrics. **A.** Distinct genes/cell from which transcripts were detected (nFeature_RNA in Seurat). The modest decline over development likely reflects the transition of a more restricted transcriptome during differentiation. **B.** Distinct UMIs recovered per cell (nCount_RNA in Seurat). **C.** Percentage of transcripts mapping to genes encoding ribosomal proteins. The increase over development reflects the maternal-to-zygotic transition in protein expression. **D.** Percentage of transcripts mapping to genes encoding cell cycle control proteins. The slight decrease in the last two stages reflects the slowing of cell division as differentiation commences in the early larva.

**
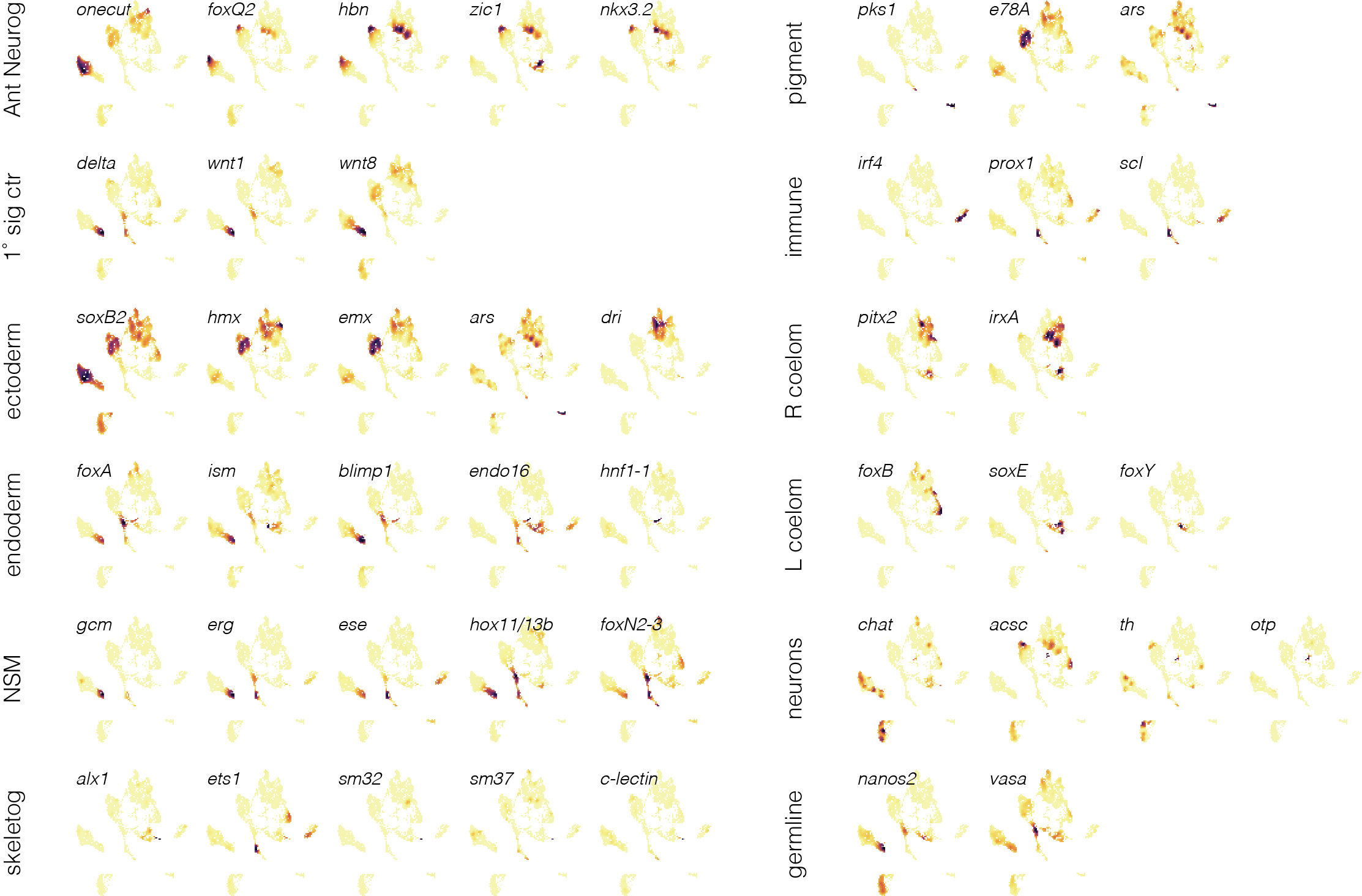
**

**Figure S2. Expression of marker genes in *H. erythrogramma.*** Density plots showing the distribution in UMAP space of cells expressing marker genes for specific embryonic territories and larval cell types. See Massri et al. 2021 for supporting literature.


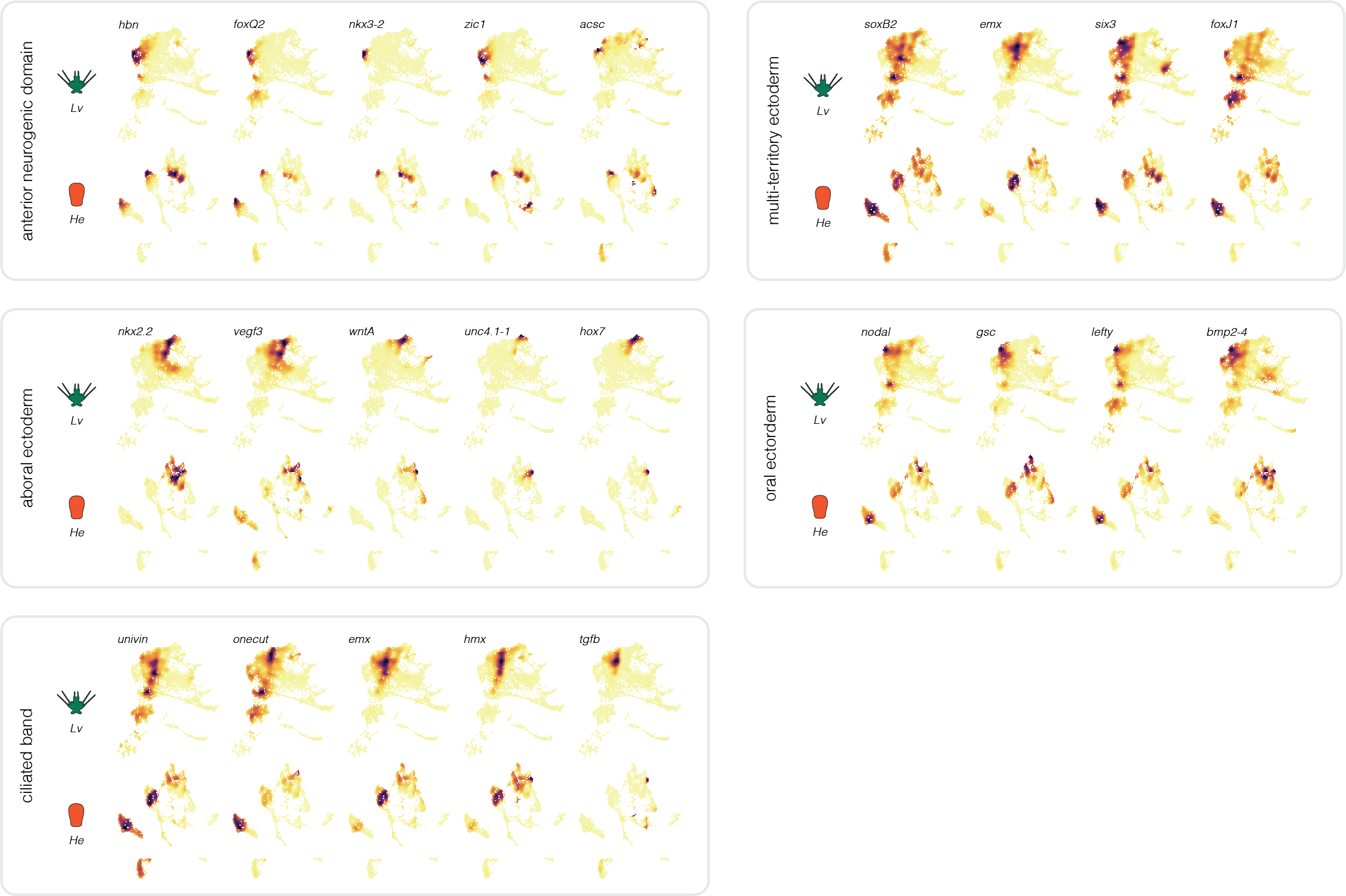


**Figure S3. Expression of marker genes for ectodermal territories in *L. variegatus* and *H. erythrogramma*.** Density plots showing the distribution in UMAP space of cells expressing marker genes for specific embryonic territories and larval cell types. See Massri et al. 2021 for supporting literature.


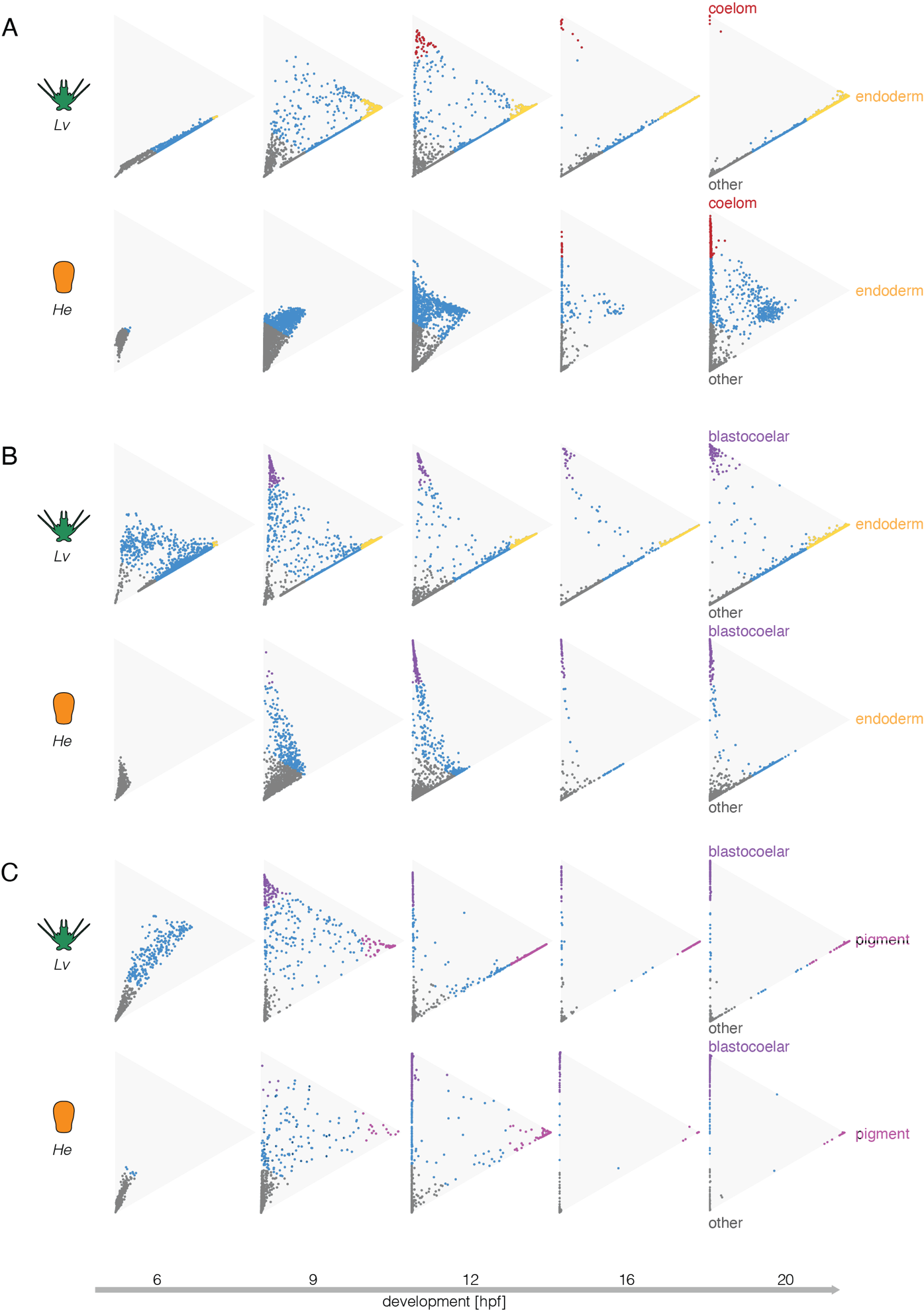


**Figure S4. Evolutionary changes in timing of differentiation, additional examples.**

Optimal transport was used to predict the likely fate for each cell at five stages, based on transcriptomes at 24 hpf (see Methods). The organization of this figure parallels that of main text Figure 4. Triangle plots show transcriptomes predictive of two specific cell types. **A.** Coelom (red) and endoderm (yellow). **B.** Blastocoelar cells (purple) and endoderm (yellow). **C.** Blastocoelar cells (purple) and pigment cells (pink). Any other cell fate is indicated in dark gray; cells with undifferentiated transcriptomes are shown in blue. See text for interpretation.


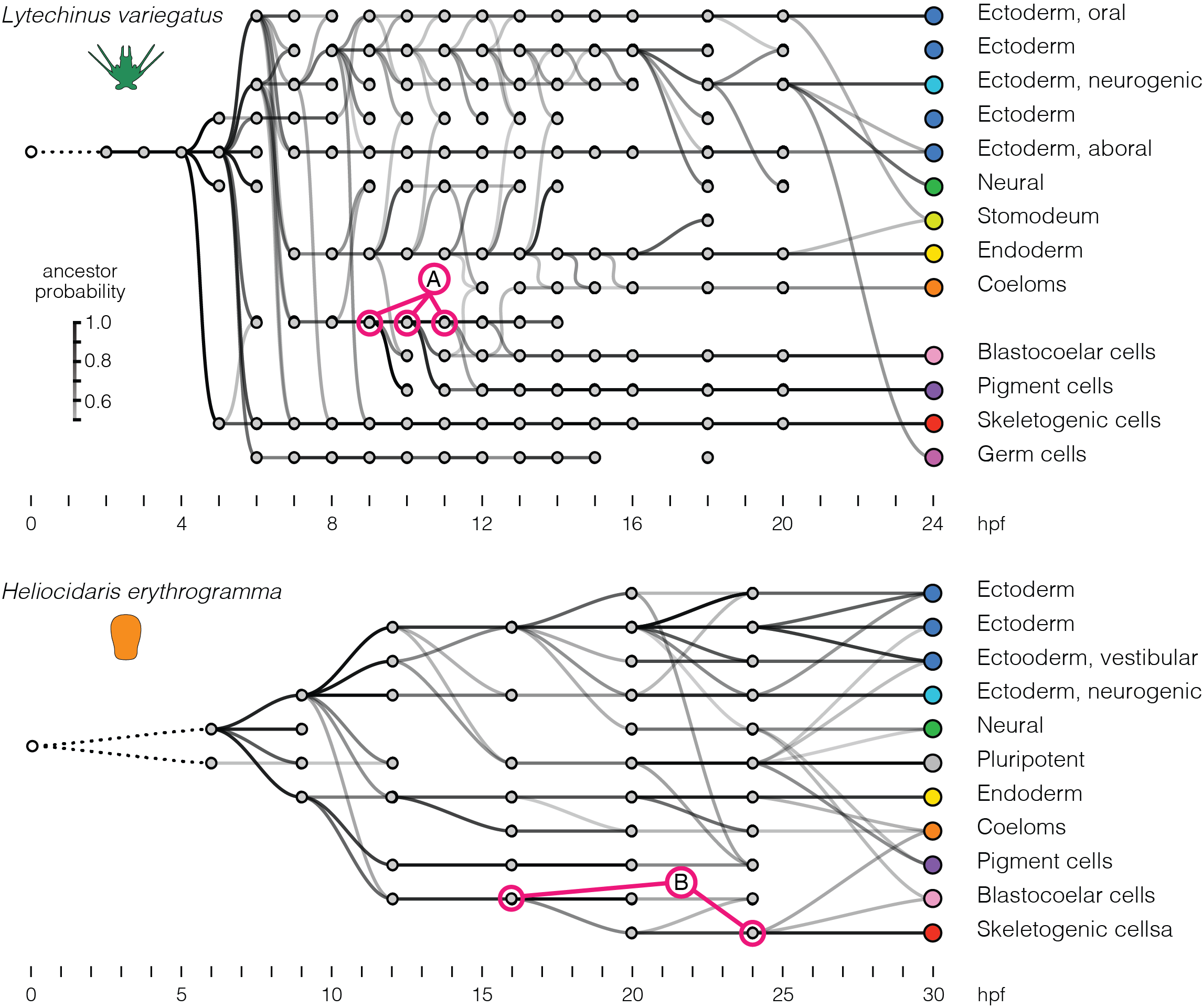


**Figure S5. Evolutionary changes in transcriptional trajectories.** Optimal transport was used to identify “ancestors” of each cell cluster at the previous time point, starting with the final time point and working backwards. (This process differs from Figures 3 and 4, where transcriptomes of individual cells, rather than clusters, are measured against those of differentiated cells). These trajectories reflect the progressive divergence of transcriptomes among cells during development, and thus are an indirect reflection of cell lineages. Top, transcriptional trajectory of *L. variegatus*; bottom transcriptional trajectory of *H. erythrogramma*. Edges are shaded to indicate the strength of inferred ancestry, with lighter tone indicating less certainty. Pink circles highlight an evolutionary difference in the topology of inferred cell lineages. **A** indicates an ancestral cell population in *L. variegatus* that gives rise to pigment cells and blastocoelar cells while excluding skeletogenic cells, consistent with published cell lineage tracing (Cameron et al. 1990; Ruffins and Ettensohn 1996; Martik and McClay 2017). **B** indicates an ancestral cell population in *H. erythrogramma* that gives rise to skeletogenic cells and blastocoelar cells while excluding pigment cells, suggesting an evolutionary difference in the order of cell fate specification events.

**
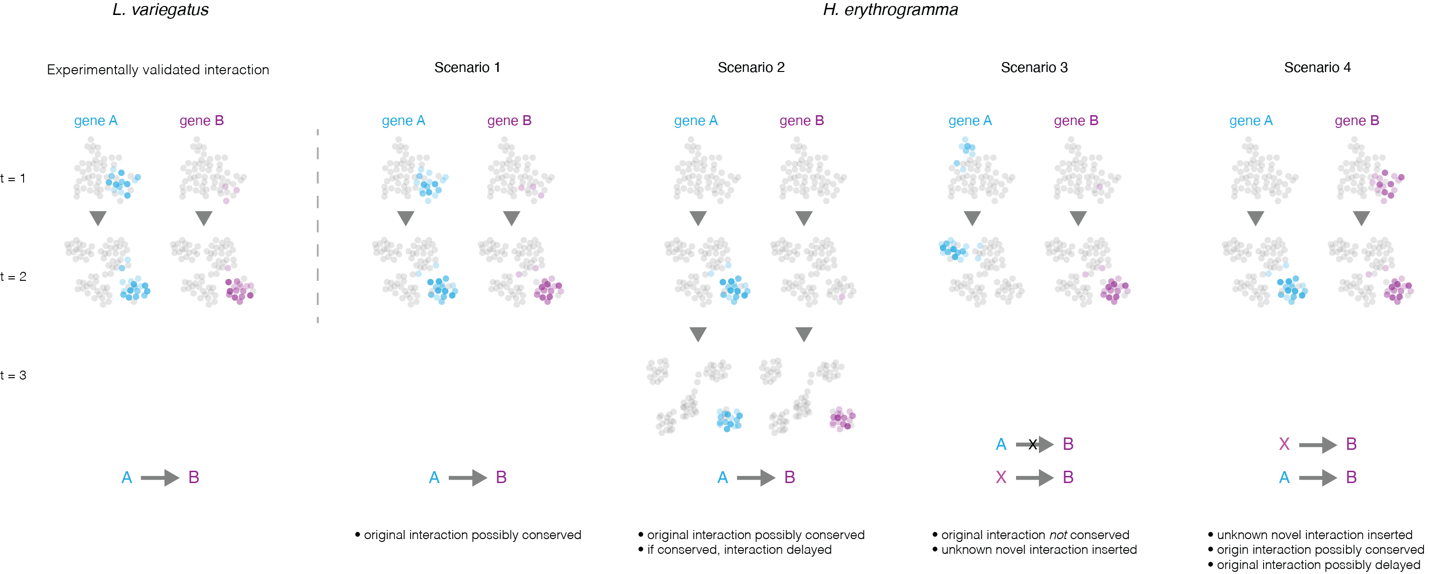
**

**Figure S6. Logic underlying inference of distinct types of evolution change in regulatory interactions.** On the extreme left, hypothetical data from *L.variegatus* illustrating an experimentally validated regulatory interaction: transcriptional activator, gene A (blue) and its target, gene B (purple), are co-expressed in the same cells. Four possible scenarios in *H.erythrogramma* for the same pair of genes are shown to the right, with interpretations regarding the regulatory interaction shown below. Scenario 1 is consistent with conservation. Scenario 2 is consistent with conservation, but with a delay in the timing of the interaction. Scenario 3 implies that the interaction no longer occurs and that some other transcription factor must activate expression of gene B. Scenario 4 is consistent with conservation, but additionally implies that some other transcription factor must operate earlier to initiate expression of gene B.

**
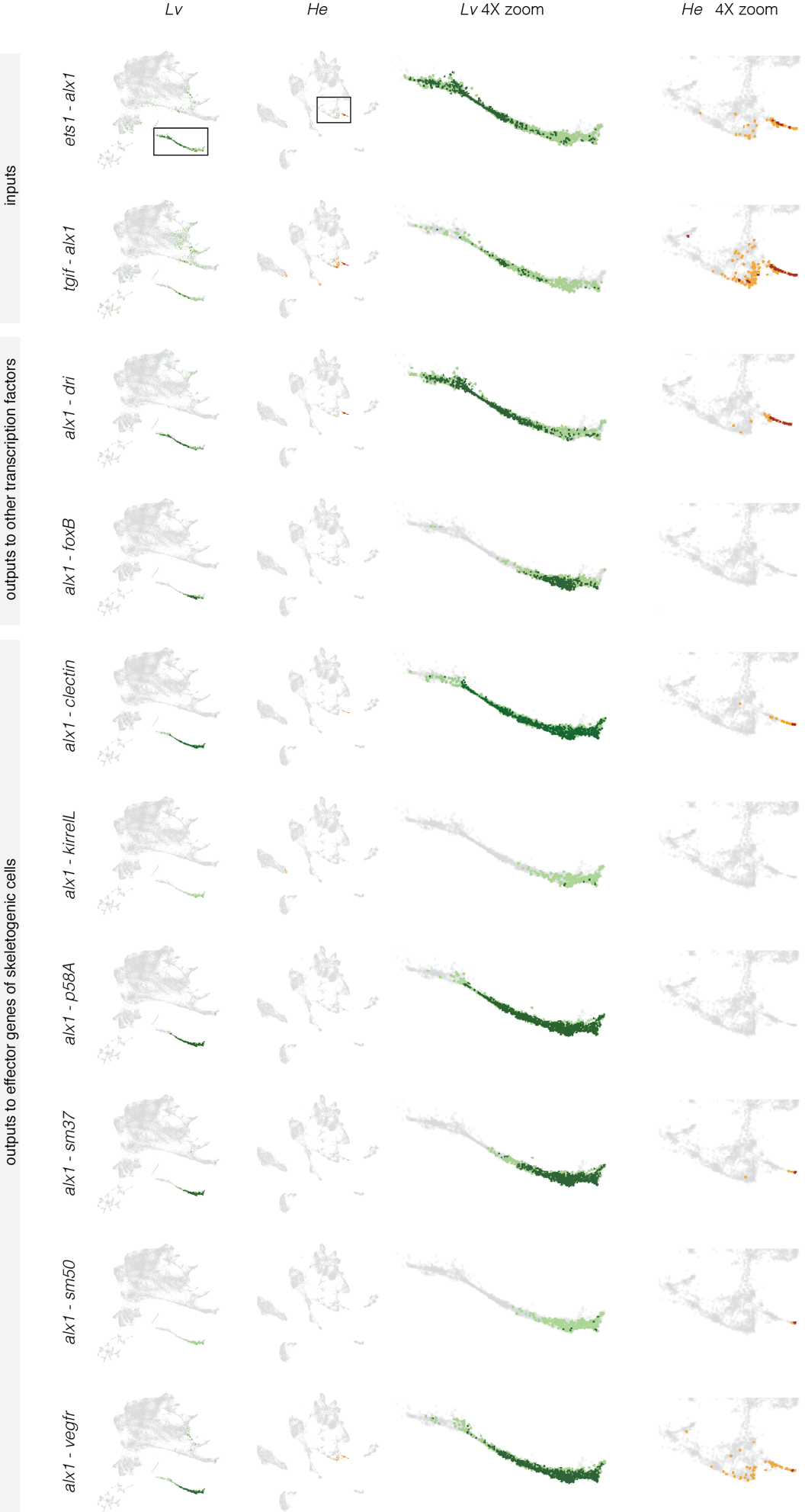
**

**Figure S7. Co-expression of *alx1* and several of its interactors within the skeletogenic dGRN.** See caption to Figure 7.

**
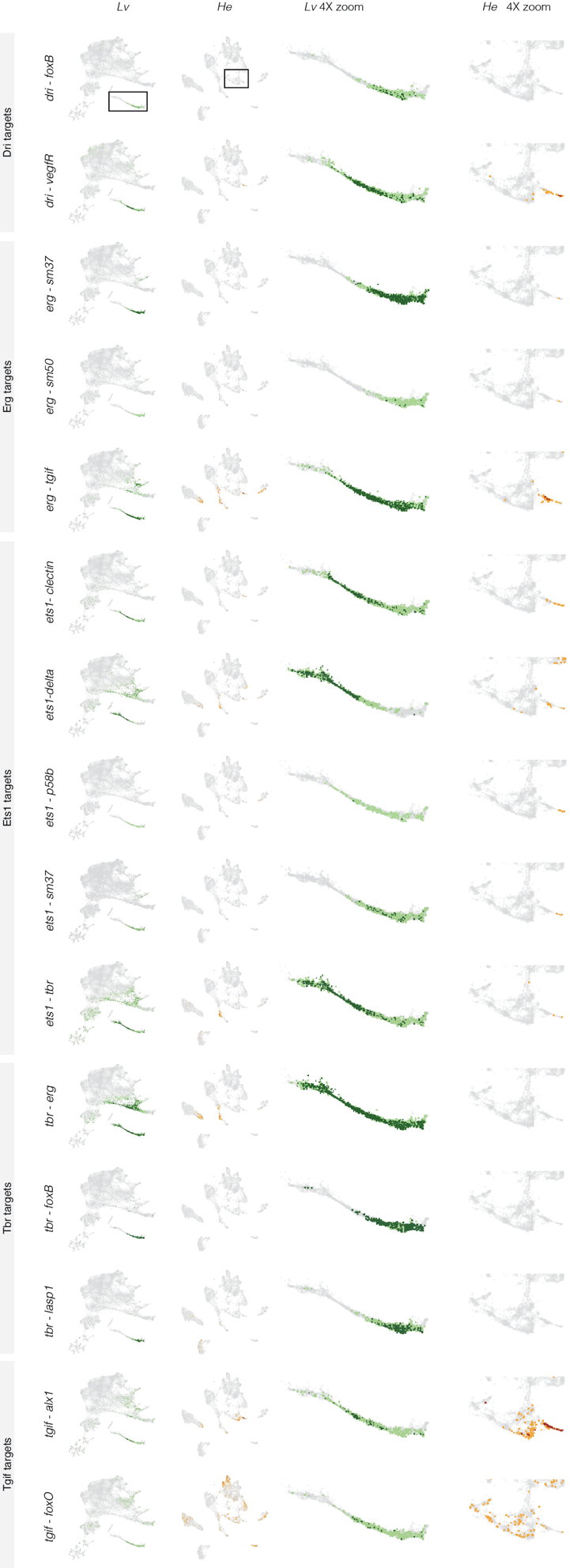
**

**Figure S8. Co-expression of additional regulators and targets within the skeletogenic dGRN.** See caption to Figure 7.

**
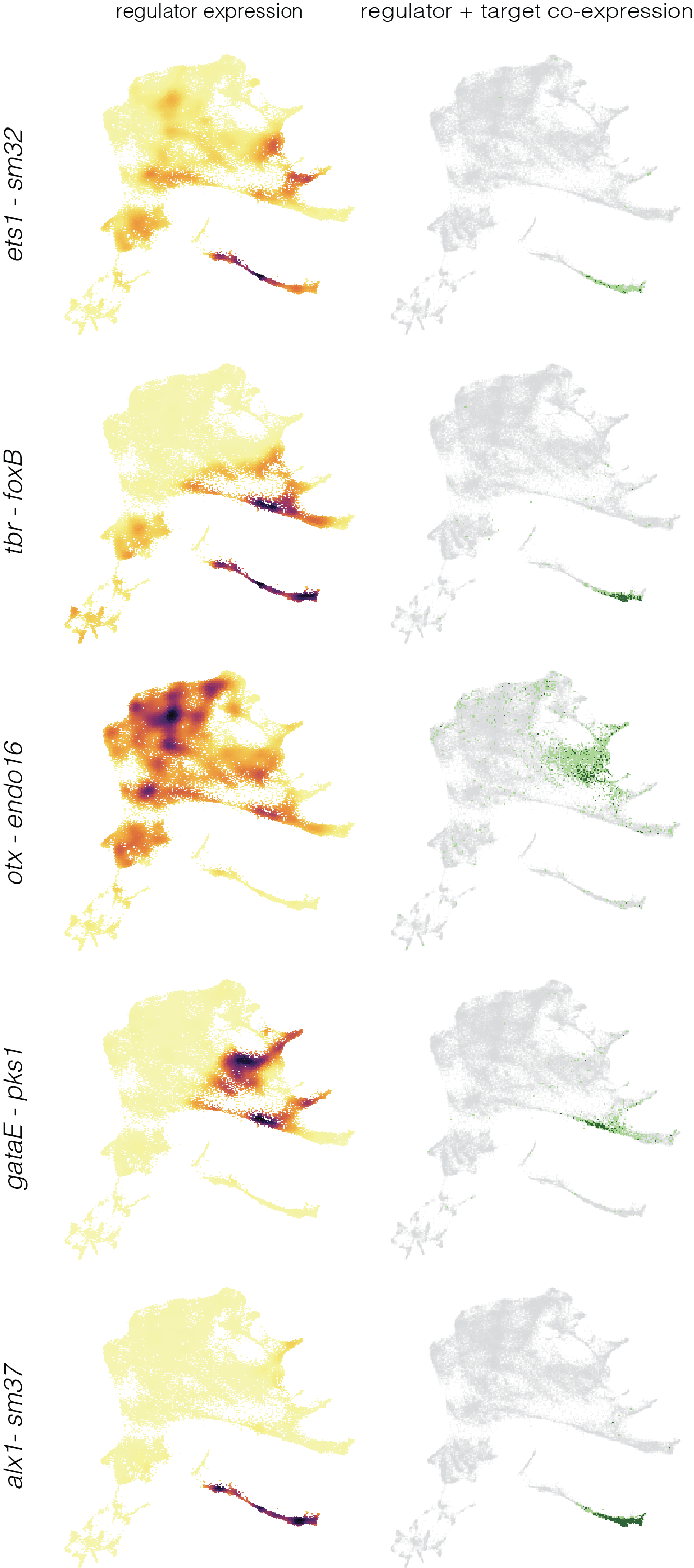
**

**Figure S9. Comparison of regulator expression and co-expression with a specific target**

**gene.** Five validated transcription factor - target gene interactions are illustrated. Left panels show density plots of expression for the regulator while right panels show cells expressing both the regulator and its target. Note that the expression domain of a transcriptional activator is often broader than its co-expression with a specific target gene.


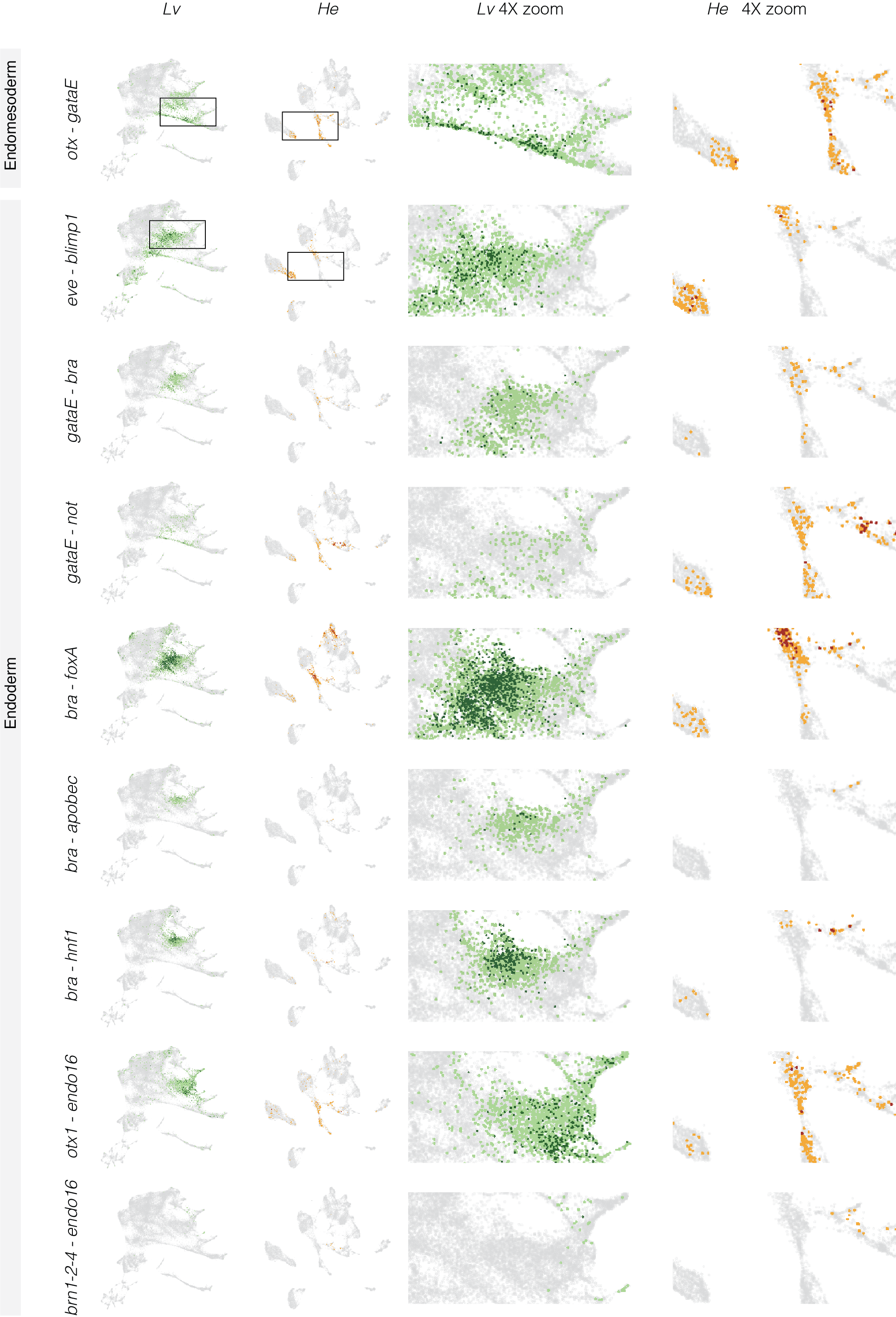


**Figure S10. Co-expression of several regulators and targets in non-skeletogenic territories of the dGRN.** See caption to Figure 7.

**
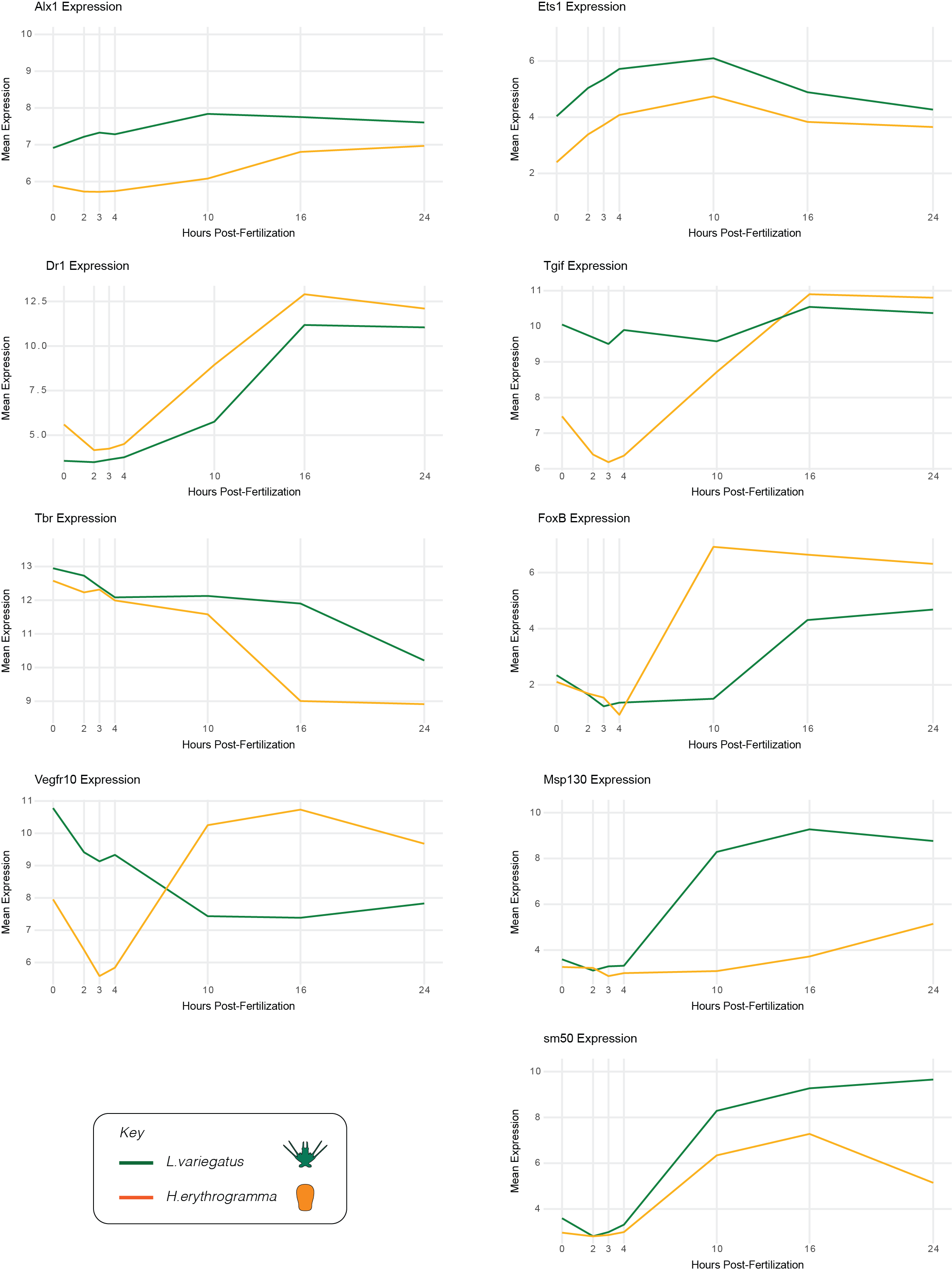
**

**Figure S11. Detailed plots of bulk RNA-seq time courses for genes shown in Figure 6B.** These bulk RNA-seq time courses provide expression values as counts normalized across time points and species as log2 counts per million + 1 (CPM+1). VLE = very low expression, here defined as <5 CPM in total across 3 biological replicates. Data from Israel et al. 2016.

**Table S1. Cell counts over development in *L. variegatus*.**

**Table S2. Cell counts over development in *H. erythrogramma*.**
